# Transgenic Expression of the piRNA-Resistant *Masculinizer* Gene Induces Female-Specific Lethality and Partial Female-to-Male Sex Reversal in the Silkworm, *Bombyx mori*

**DOI:** 10.1371/journal.pgen.1006203

**Published:** 2016-08-31

**Authors:** Hiroki Sakai, Megumi Sumitani, Yasuhiko Chikami, Kensuke Yahata, Keiro Uchino, Takashi Kiuchi, Susumu Katsuma, Fugaku Aoki, Hideki Sezutsu, Masataka G. Suzuki

**Affiliations:** 1 Department of Integrated Biosciences, Graduate School of Frontier Sciences, The University of Tokyo, 5-1-5 Kashiwanoha, Kashiwa-shi, Chiba 277-8562, Japan; 2 Transgenic Silkworm Research Unit, Institute of Agrobiological Sciences, National Agriculture and Food Research Organization (NARO), Owashi, Tsukuba, Ibaraki 305-8634, Japan; 3 Graduate School of Life and Environmental Sciences, University of Tsukuba, Tsukuba, Ibaraki 305-8572, Japan; 4 Faculty of Life and Environmental Sciences, University of Tsukuba, Tsukuba, Ibaraki 305-8572, Japan; 5 Department of Agricultural and Environmental Biology, Graduate School of Agricultural and Life Sciences, The University of Tokyo, 1-1-1 Yayoi, Bunkyo-ku, Tokyo 113-8657, Japan; University of California Davis, UNITED STATES

## Abstract

In *Bombyx mori* (*B*. *mori*), *Fem* piRNA originates from the W chromosome and is responsible for femaleness. The *Fem* piRNA-PIWI complex targets and cleaves mRNAs transcribed from the *Masc* gene. *Masc* encodes a novel CCCH type zinc-finger protein and is required for male-specific splicing of *B*. *mori doublesex* (*Bmdsx*) transcripts. In the present study, several silkworm strains carrying a transgene, which encodes a *Fem* piRNA-resistant *Masc* mRNA (*Masc-R*), were generated. Forced expression of the *Masc-R* transgene caused female-specific lethality during the larval stages. One of the *Masc-R* strains weakly expressed *Masc-R* in various tissues. Females heterozygous for the transgene expressed male-specific isoform of the *Bombyx* homolog of insulin-like growth factor II mRNA-binding protein (*Imp*^*M*^) and *Bmdsx*. All examined females showed a lower inducibility of vitellogenin synthesis and exhibited abnormalities in the ovaries. Testis-like tissues were observed in abnormal ovaries and, notably, the tissues contained considerable numbers of sperm bundles. Homozygous expression of the transgene resulted in formation of the male-specific abdominal segment in adult females and caused partial male differentiation in female genitalia. These results strongly suggest that *Masc* is an important regulatory gene of maleness in *B*. *mori*.

## Author Summary

In the silkworm, *Bombyx mori*, a W-chromosome-linked gene *Feminizer* (*Fem*) determines femaleness. *Fem* transcript yields a piRNA (*Fem* piRNA) and *Fem*- piRNA-PIWI complex targets and cleaves mRNAs transcribed from the *Masculinizer* (*Masc*). *Masc* is required for male-specific expression of *Bmdsx*, which is an important regulatory gene for sexual differentiation, and therefore, *Masc* is considered to be essential for maleness. However, there has been no direct evidence that *Masc* indeed causes maleness in sexually dimorphic structures. To clarify this point, we established silkworm strains carrying a transgene that expresses *Fem*-piRNA-resistant *Masc* gene (*Masc-R*). Transgenic expression of the *Masc-R* induced male mode of expressions of downstream sex-determining genes in females. Notably, ovaries in these females exhibited testis-like structures that contained sperm bundles. Homozygous expression of the *Masc-R* caused formation of the male-specific abdominal segment in adult females and induced partial male differentiation in female genitalia. Thus, *Masc* can induce maleness at the morphological level and is sufficient for spermatogenesis. This is the first report to our knowledge on a gene that can masculinize a wide variety of sexual characteristics in lepidopteran insects.

## Introduction

Most animal species have two sexes and display various sexual dimorphisms. Mechanisms of sex determination are highly different among phyla [1–3]. In many reptile species, the temperature at which eggs are incubated determines their sex [4]. In *Daphnia magna*, a shortening of the photoperiod, lack of food, and increase in population density leads to the production of males that are genetically identical to females [5]. Mammals show genotypic sex determination, with sex determined by the expression of a Y-linked gene, *Sry* [6]. In the African clawed frog, *Xenopus laevis*, DM-W located on the W chromosome induces female development [7].

Given the different sex-determining switches among species, genes from the Doublesex Mab-3 Related Transcription (DMRT) family are highly conserved as components of the vertebrate and invertebrate sex-determining pathways [8]. In insects, *doublesex* (*dsx*) is a well known *Dmrt* family gene. The *dsx* gene, which produces female- and male-specific transcripts by sex-specific alternative splicing, and is situated at the bottom of the sex determination cascade, has been reported in various insects [9]. In *Drosophila melanogaster*, female-specific splicing of *dsx* requires both TRA, a protein product of *transformer* (*tra*) whose functional isoform is produced only in females, and a protein product of *transformer-2* (*tra-2*) [10]. A similar function of *tra* has been reported in several dipteran, hymenopteran, and coleopteran insects. RNA interference (RNAi)-mediated knockdown of *tra* in *Musca* [11], *Ceratitis* [12], *Lucilia* [13], *Nasonia* [14], *Bactrocera* [15], and *Tribolium* [16] caused male-specific splicing of the endogenous *dsx* pre-mRNAs, leading to masculinization of chromosomal females. Therefore, *tra* has been considered a consensus upstream regulator that directs female-specific splicing of *dsx* transcripts in insects. This hypothesis has now been challenged because *tra* is absent in several species belonging to four insect orders: Lepidoptera, basal Diptera, Strepsiptera, and Coleoptera [17]. A *tra* ortholog has not been identified in the silkworm, *Bombyx mori* (*B*. *mori*), which is a model organism of lepidopteran insects. The female exon of the *Bombyx* homolog of *dsx* (*Bmdsx*) is devoid of putative TRA-TRA2-binding sites [18]. Moreover, RNAi knockdown of the *Bombyx* ortholog of *tra*-2 does not influence sex-specific splicing of *Bmdsx* pre-mRNA [19]. However, the male splicing of *Bmdsx* transcripts requires the splicing inhibitor (BmPSI) and the male-specific isoform of the *Bombyx* homolog of insulin-like growth factor II, mRNA-binding protein (IMP^M^). These proteins form a complex that binds to a *cis*-regulatory element called CE1, located in the female-specific exon, and inhibit the female mode of splicing in males [20,21]. These results support that the sex determination cascade of *B*. *mori* is different from the already known sex determination cascade.

The femaleness of *B*. *mori* is predominantly determined by the presence of the W chromosome and a master regulatory gene at the top of the sex determination cascade thought to exist on the W chromosome [22]. We recently identified a W-linked gene, *Feminizer* (*Fem*), which is the master regulatory gene for femaleness. *Fem* transcripts yield PIWI-interacting RNAs (piRNAs), which are designated as *Fem* piRNAs and are responsible for femaleness [23]. piRNAs are 23–30 nucleotide (nt)-long small RNAs that act as sequence-specific guides for PIWI proteins that cleave target RNAs, mainly to disrupt the activity of transposons in gonads [24]. *Fem* piRNA-PIWI protein complex targets and cleaves mRNAs transcribed from the Z chromosome-linked gene that encodes a CCCH-tandem zinc-finger protein [23]. Knockdown of the expression of this gene in male embryos leads to the production of the female-type isoform of *Bmdsx* [23] and decreases the expression level of *Imp*^*M*^ [25]. These results demonstrate that this Z-linked gene is essential for silkworm masculinization, which was named *Masculinizer* (*Masc*). However, there has been no report showing that knockdown of *Masc* causes morphological changes in sexually dimorphic structures because knockdown of *Masc* causes male-specific embryonic lethality presumably due to defects in the gene dosage compensation of Z-linked genes [23].

To examine whether *Masc* controls male development, we performed ectopic expression analysis of *Masc* using transgenic silkworm strains. For this purpose, we created a transgene designed to allow expression of *Fem* piRNA-resistant *Masc* cDNA (*Masc-R*) under the control of the GAL4-UAS binary expression system [26]. Our results showed that forced expression of *Masc-R* induced female-specific lethality during the larval stage. Moreover, expression of *Masc-R* in females strongly repressed femaleness expressed in vitellogenin synthesis, egg production, and ovary formation and, surprisingly, caused spermatogenesis in a testis-like tissue ectopically formed in the ovary. Thus, *Masc* can induce maleness at the morphological level and is sufficient for spermatogenesis.

## Results

### Establishment of transgenic silkworms expressing *Masc-R*

**Fig 1:**
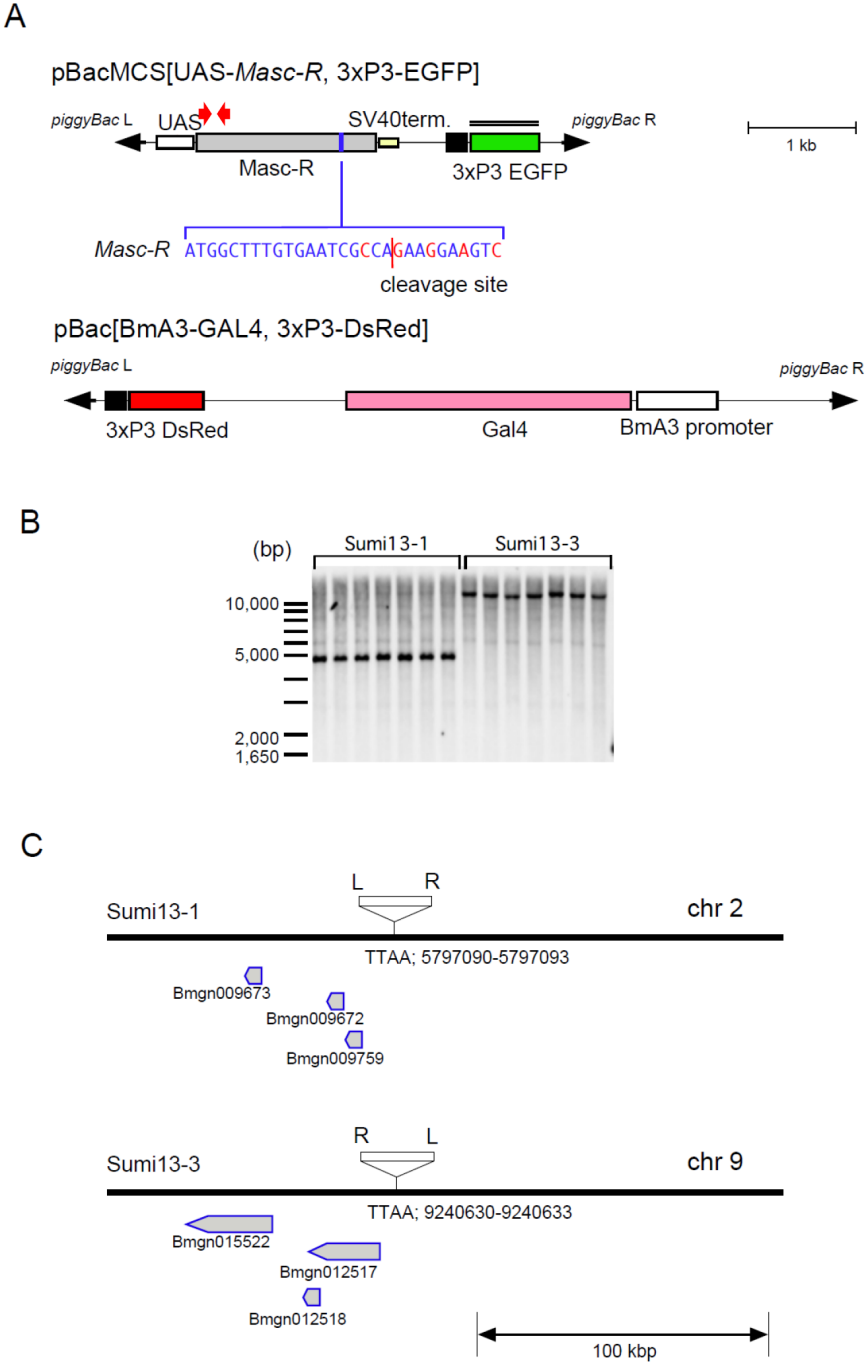
Generation of transgenic silkworms that express *Masc-R*. (A) Construction of the *piggyBac* transformation vector, pBacMCS [UAS-*Masc-R*, 3×P3-EGFP] (in this study) and pBac [BmA3-GAL4, 3×P3-DsRed][27]. Grey box represents the open reading frame of *Masc-R*. Target region by *Fem* piRNA is shown by the blue box, and the sequences are displayed. *Masc-R* contains 5 nucleotide substitutions (red characters) from the wild-type sequence, which makes it resistant to cleavage by the *Fem* piRNA-PIWI protein complex. The red line represents the cleavage site. *piggyBac* inverted terminal repeats are shown as block arrows. UAS, upstream activation sequence; SV40 term, 3' untranslated region of Simian virus 40 including polyadenylation signal for the termination. The double line indicates the position of the probe used in the Southern blot analysis. The red arrows indicate the approximate location of the primers used for RT-PCR analysis described in Fig 2A. (B) Southern blot analysis of two transgenic strains (Sumi13-1 and 13–3). Genomic DNA prepared from transgenic lines was digested with *Bam*HI and analyzed with a probe containing the EGFP sequence. The molecular size is shown on the left side of the membranes. (C) Schematic diagram of the insertion site of the transgene in each transgenic strain (Sumi13-1 and 13–3) revealed by inverse-PCR. Neighboring genes that exist around the insertion site are displayed. White box indicates the transformation vector. *piggyBac*L and R arms are shown as L and R, respectively. The position number of the TTAA insertion sequence within the chromosome is described below the line.

As reported previously, forced expression of *Masc* cDNA in *B*. *mori* ovary-derived cell line (BmN4) induces male-specific variants of *Bmdsx*, but the masculinizing activity was relatively low due to the cleavage of *Masc* mRNA in the presence of *Fem* piRNA in female cells [23]. To overcome this problem, we utilized *Masc-R* cDNA (Fig 1A) that is resistant to *Fem* piRNA-mediated cleavage [23]. In this study, *Masc-R* cDNA was expressed using a GAL4-upstream activation sequence (GAL4-UAS) system specifically arranged for the silkworm [26]. Structure of the transformation vector carrying UAS-*Masc-R* was illustrated in Fig 1A. The resultant transgenic lines (Sumi13-1 and 13–3) were viable and fertile; however, fertility of Sumi13-3 was very low. Southern blot analysis revealed that the UAS-*Masc-R* sublines carried only one copy of the transgene (Fig 1B). Inverse PCR analyses showed that the UAS-*Masc-R* transgenes in Sumi13-1 and 13–3 were integrated into an autosomal region within chromosome 2 and chromosome 9, respectively. As shown in Fig 1C, the insertions did not disrupt gene structures present near the insertion site.

### Forced expression of *Masc-R* induced female-specific lethality during the larval stage

We examined the effects of *Masc-R* expression on female development. For this purpose, we crossed Sumi13-1 and Sumi13-3 males heterozygous for the UAS-*Masc-R* transgene with females that ubiquitously express GAL4 through *B*. *mori cytoplasmic actin 3* promoter (BmA3-GAL4). The BmA3-GAL4 strain was maintained heterozygous for the BmA3-GAL4 transgene. The strain expressed DsRed as a selection marker, which enables identification of genotype by visualizing fluorescence in the eye.

**Fig 2:**
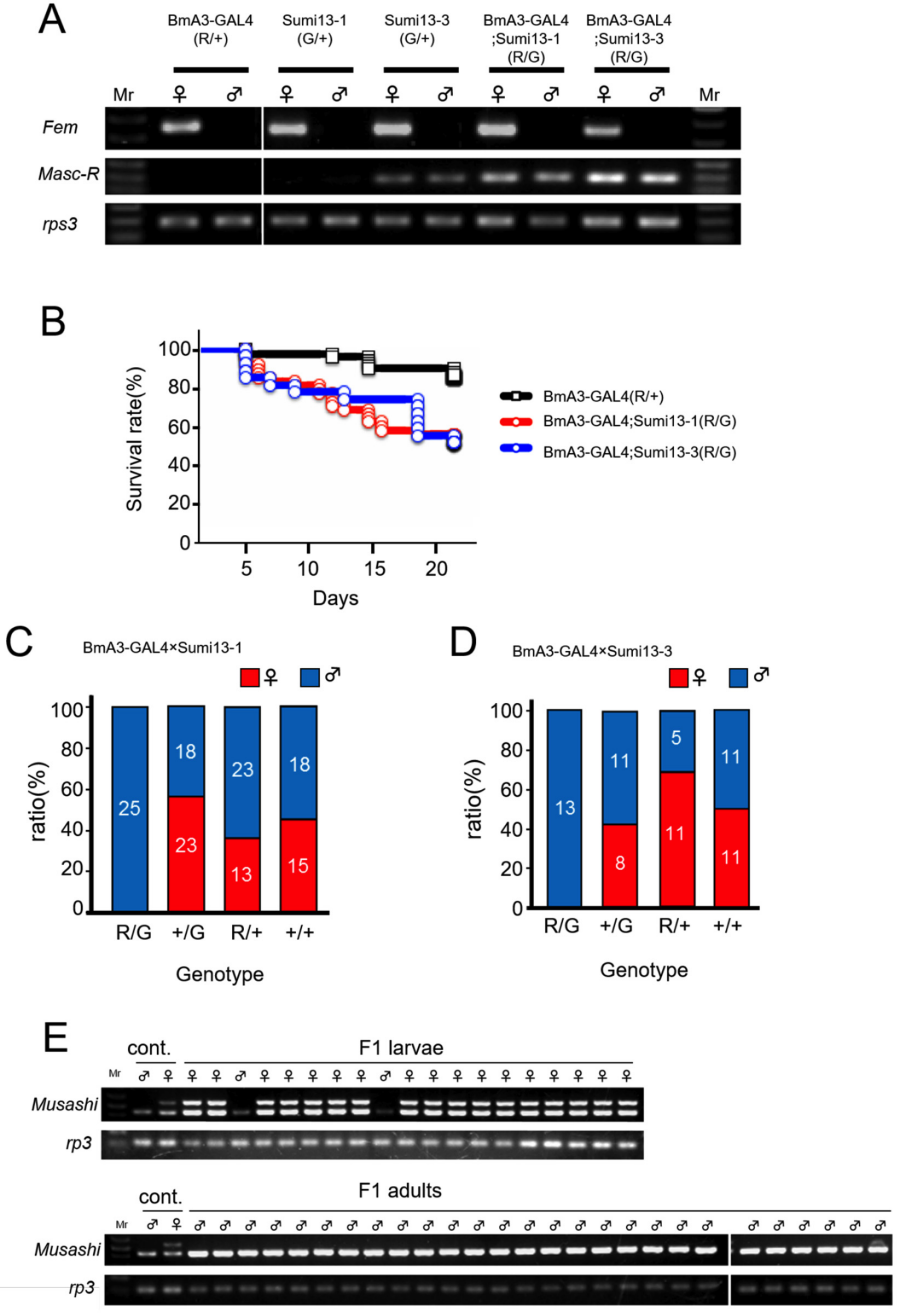
GAL4-UAS mediated expression of *Masc-R* caused severe lethality in females. (A) Forced expression of *Masc-R* utilizing the GAL4-UAS system in the transgenic strains. The expression of *Masc-R* mRNA in first instar larvae just after hatching was detected by RT-PCR. Individual sex was determined by the expression of *Fem* RNA. The universally expressed *ribosomal protein S3* (*rpS3*) was used as an internal control. Fluorescent-positive animals expressing either DsRed (R / +), EGFP (G / +), or both (R / G) were examined. R / + and G / + animals carried either BmA3-GAL4 or UAS-*Masc-R* transgenes, respectively. R / G animals possessed both BmA3-GAL4 and UAS-*Masc-R*. (B) Kaplan—Meier survival curve was plotted with survival rate (%) on *Y*-axis and the number of days after hatching on the *X*-axis. BmA3-GAL4 (R/+) n = 68, BmA3-GAL4; Sumi13-1 (R/G) n = 48, BmA3-GAL4; Sumi13-3 (R/G) n = 27, P = 0.00013 [BmA3-GAL4 (R/+) versus BmA3-GAL4; Sumi13-1 (R/G)], p = 0.00020 [BmA3-GAL4 (R/+) versus BmA3-GAL4; Sumi13-3(R/G)], log rank test. (C)(D) The sex ratio of F1 adult moths obtained by crossing BmA3-GAL4 females either with UAS-*Masc-R* (Sumi13-1 and Sumi13-3) males (C and D). Blue and red bars indicate the number of males and the number of females, respectively. (E) The sexing of F1 individuals obtained by crossing BmA3-GAL4 females with UAS-*Masc-R* males (Sumi13-1). PCR-amplification of *Musashi* [28], a marker gene on the W chromosome, using genomic DNA isolated from F1 dead larvae (upper panel) or animals that reached the adult stage (lower panel). Two different sizes of DNA fragments were amplified in females, whereas the same PCR reactions detected only a single DNA fragment in males. Based on the size of the amplified products, upper band corresponded to the W-chromosome specific marker *Musashi*, whose size is 574 bp [28]. The smaller DNA fragment amplified in both males and females was assumed to be a non-specifically amplified DNA derived from autosome.

The hatch rate of F1 embryos derived from each crossing (BmA3-GAL4 × Sumi13-1 and BmA3-GAL4 × Sumi13-3) was similar to that of control embryos (Table 1). These results demonstrated that forced expression of *Masc-R* did not affect embryogenesis. The number of F1 individuals at the fifth instar larvae, and the adult stages in each crossing, were counted. F1 individuals expressing the UAS-*Masc-R* transgene were obtained by selecting double fluorescent-positive animals that expressed both EGFP and DsRed (S1 Fig). Reverse transcription (RT)-PCR analyses confirmed that forced expression of *Masc-R* occurred in the double fluorescent-positive animals (Fig 2A). Interestingly, Sumi13-3 animals weakly expressed *Masc-R* independent of GAL4 induction. The survival rate of *Masc-R*-expressing animals (genotype R/G in both BmA3-GAL4 × Sumi13-1 crossing and BmA3-GAL4 × Sumi13-3 crossing) was significantly lower than that of GAL4-expressing animals with other genotypes (BmA3-GAL4) (Fig 2B). The survival curves shown in Fig 2B indicated that half of *Masc-R*-expressing animals were dead before the third instar larval stage. Notably, surviving animals expressing UAS-*Masc-R* transgene were all males (genotype R/G in Fig 2C and genotype R/G in Fig 2D), while the sex ratio of animals with all other genotypes (animals without *Masc-R* expression) was approximately 50% (genotype R/+, G/+, +/+ in Fig 2C and genotype R/+, G/+, +/+ in Fig 2D). These results strongly suggested that *Masc-R* expression caused female-specific lethality during larval and pupal development. To confirm this hypothesis, we determined the sex of all *Masc-R*-expressing individuals (including dead ones) by PCR with primers that specifically amplify the genomic region located on the W chromosome. The PCR-based sexing demonstrated that all surviving adult moths were male and that 89% of the dead larvae were female (Fig 2E). No surviving males showed any abnormalities in sexual dimorphisms. These results indicated that forced expression of *Masc-R* caused female-specific lethality during larval stages.

**Table 1:** Number of hatching and viable F1 individuals in each crosses.

Cross	Genotype of F1	No. of embryos	No. of embryos hatching	Hatching rate (%)	No. of 5th instar larvae normally developed	
					♀	♂
BmA3-GAL4×Sumi13-1	R/+; G/+	49	48	98	0	27
	G/+	50	49	98	23	21
	R/+	44	42	95	16	24
	+/+; +/+	37	37	100	15	20
BmA3-GAL4×Sumi13-3	R/+; G/+	28	27	96	0	14
	G/+	32	32	100	9	18
	R/+	31	30	97	20	10
	+/+; +/+	26	25	96	12	12

R/+ (DsRed); G/+ (EGFP) animals possessed both BmA3-GAL4 and UAS-*Masc-R*.

R/+ and G/+ animals carried either BmA3-GAL4 or UAS-*Masc-R* transgenes, respectively.

+/+; +/+ animals had no transgenes.

### Expression of *Masc-R* in females causes ectopic expression of male forms of *Imp* and *Bmdsx*

We next investigated the splicing pattern of *Bmdsx* in *Masc-R*-expressing females when they were still alive (S2 Fig). In addition to the female form of *Bmdsx* transcripts (*BmdsxF*), male form of *Bmdsx* transcripts (*BmdsxM*) was obviously expressed in females that possessed both BmA3-GAL4 and UAS-*Masc-R* (S2 Fig). This result indicated that forced expression of *Masc-R* was able to shift the splicing pattern of *Bmdsx* from female to male mode. However, we could not further investigate the role of *Masc* in sexual differentiations morphologically using this system because BmA3-GAL4-induced expression of *Masc-R* caused severe lethality in females. We observed that Sumi13-3 animals weakly expressed the *Masc-R* gene independent of GAL4 induction (Fig 2A), and thus used this line in further experiments. Silkworm strain Suzu19-1 was used to discriminate females from males. This strain has a W-chromosome-linked transgene that carries a DsRed gene under the control of 3×P3 unit. We crossed Sumi13-3 males heterozygous for the UAS-*Masc-R* transgene with Suzu19-1 females. We selected the progenies which expressed DsRed and EGFP. The progenies were heterozygous for the UAS-*Masc-R* transgene females (hereafter described as *Masc-R*/+ females). Although *Masc-R*/*Masc-R* females were also subjected to the analysis, it was extremely difficult to obtain *Masc-R*/*Masc-R* females as described in Materials and Methods, Therefore, in this study, *Masc-R*/+ females were mainly subjected to the following analyses.

**Fig 3:**
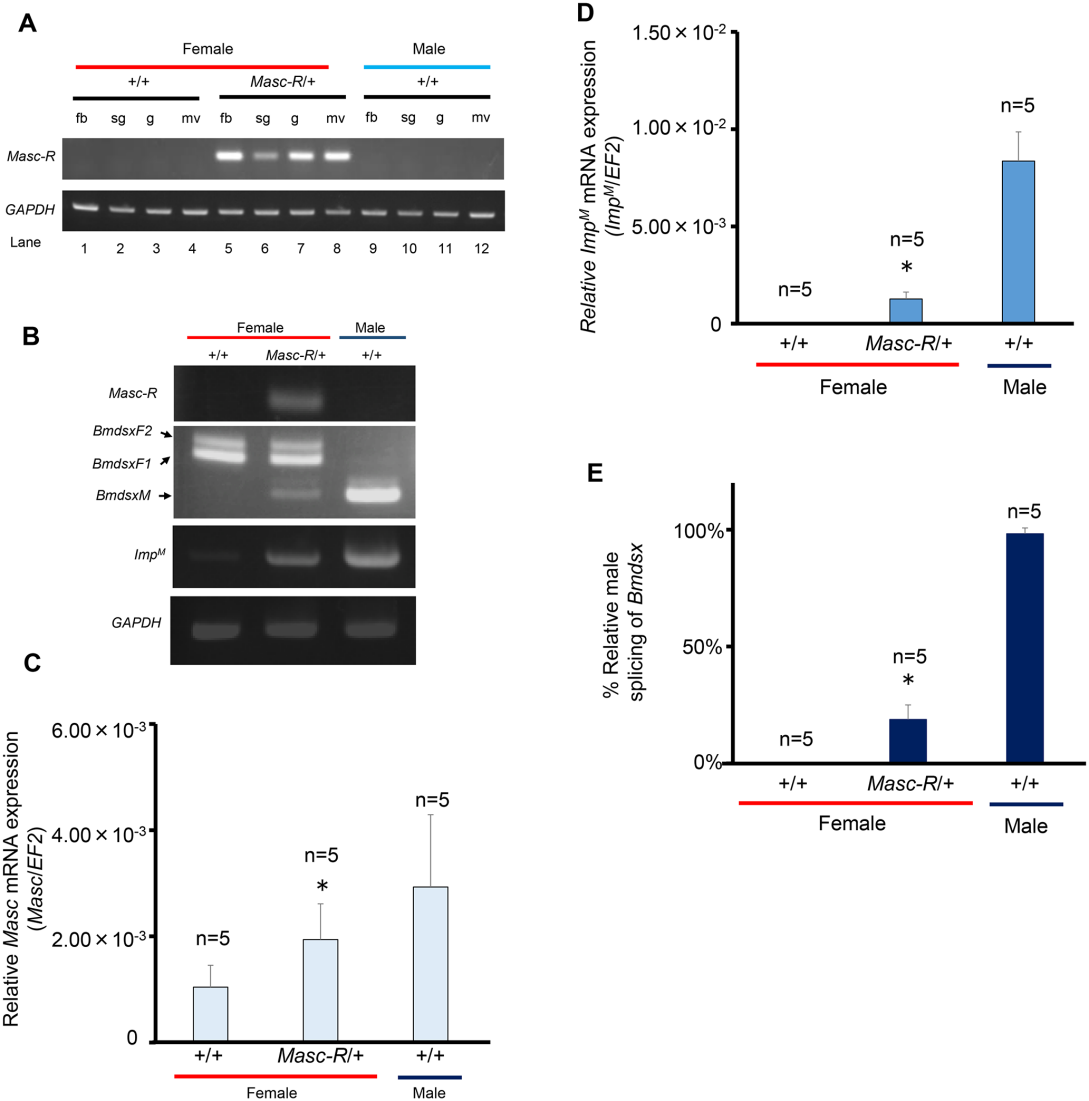
Expression patterns of sex-determining genes in Sumi13-3 females. (A) The tissue distribution of *Masc-R* expression was analyzed by RT-PCR in L5D3 (day 3 of the fifth larval instar). *Masc-R*/+, Sumi13-3 females heterozygous for the UAS-*Masc-R* transgene; +/+, Sumi13-3 sister females, which did not have the UAS-*Masc-R* transgene; fb, fat body; sg, silk gland; g, gonad; mv, malpighian vessel. (B) Expression patterns of *Masc-R*, *Bmdsx* and *Imp*^*M*^ were analyzed by RT-PCR in L1D1 (day 1 of the first larval instar). Amplified products were separated by 1% agarose gel electrophoresis. The top panel indicates *Masc-R* expression. The second panel from the top indicates the female- and male-specific splice variants of *Bmdsx* (*BmdsxF1*, *BmdsxF2* and *BmdsxM*, respectively). The third panel shows *Imp*^*M*^ expression. The bottom panel shows amplification of the *GAPDH* transcript, which served as a positive control for RNA extraction and RT-PCR. (C) Quantification of *Masc* mRNA at L1D1 and (D) Quantification of *Imp*^*M*^ mRNA at L1D1 using qRT-PCR. *EF2* was served as an internal standard. Error bar: SD; * significant differences at the 0.05 level (Welch’s t-test) compared with the +/+ female. (E) The percentage of RT-PCR products representing the male *Bmdsx* splice variant relative to the sum of the female- and male-specific *Bmdsx* splice variants. DNA bands were quantified using Image J software (ver. 1.47’ National Institutes of Health, Bethesda, MD, USA [http://rsb.info.nih.gov/ij/]). Error bar: SD. * significant differences at the 0.05 level (Mann—Whitney U test) compared with the +/+ female.

RT-PCR analyses demonstrated that the *Masc-R* gene was expressed in several organs dissected from the larvae of *Masc-R*/+ females (Fig 3A and 3B). Quantitative real-time RT-PCR (qRT-PCR) using primers that can amplify cDNAs from both endogenous *Masc* and the *Masc-R* transgene revealed a significant increase in the total expression level of *Masc* mRNA in *Masc-R*/+ females as compared with that in sister females, which did not have the UAS-*Masc-R* transgene (hereafter described as +/+ females) (Fig 3C).

Having demonstrated that expression of the *Masc-R* gene induces *Imp*^*M*^ and *BmdsxM* in BmN4 cells [29], we next investigated the expression of *Imp*^*M*^ and the splicing pattern of *Bmdsx* in *Masc-R*/+ females. RT-PCR analysis with cDNAs prepared from day-1 first instar larvae showed that *Imp*^*M*^ expression was induced in *Masc-R*/+ females (Fig 3B). Notably, qRT-PCR analysis demonstrated that the expression level of *Imp*^*M*^ was more than 28-fold higher in *Masc-R*/+ than in +/+ females (Fig 3D). In addition, the male-specific splice variant of *Bmdsx* was detected in *Masc-R*/+ females, while the female form of *Bmdsx* transcripts alone was observed in +/+ females (Fig 3B). The percentage of the male-specific splicing of *Bmdsx* relative to the total splicing of *Bmdsx* was > 18% in *Masc-R*/+ females (Fig 3B and 3E). These results indicated that expression of *Masc-R* in females caused ectopic expression of male forms of *Imp* and *Bmdsx*.

### Expression of *Masc-R* severely diminished vitellogenin synthesis in the female fat body

**Fig 4:**
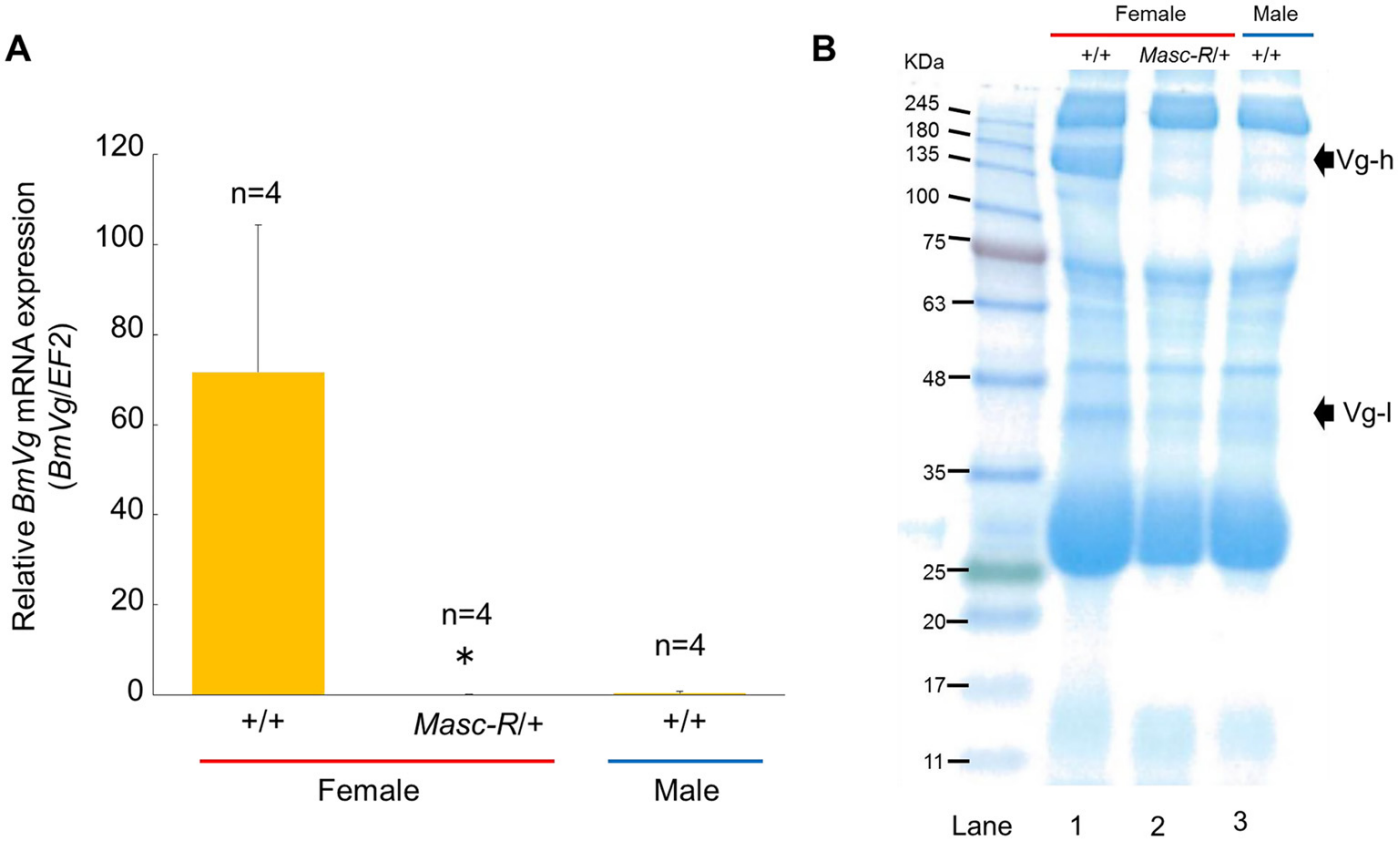
Expression of *Masc-R* severely diminished vitellogenin synthesis in the female fat body. (A) Quantification of the *BmVg* mRNA level within 3 hours after pupation using qRT-PCR. *EF2* served as an internal standard. Error bar: SD; * significant differences at the 0.05 level (Welch’s t-test) compared with the +/+ female. (B) SDS-PAGE analysis of the whole hemolymph within 3 hours after pupation. Hemolymph sample applied in each lane was a mixture of hemolymph collected from three individuals. The arrows indicate the protein bands corresponding to the molecular weight of BmVg heavy chain (Vg-h) and BmVg light chain (Vg-l), respectively.

Vitellogenins are precursors of the major yolk proteins in insects. In the silkworm, vitellogenins are predominantly synthesized in the female fat body during larval—pupal ecdysis [30,31]. RT-PCR analyses demonstrated that the expression of *Masc-R* in females caused ectopic expression of male forms of *Imp* and *Bmdsx* in fat bodies within 3 hours after pupation (S3A Fig). To investigate whether the expression of *Masc-R* affects the expression of the vitellogenin gene (*BmVg*) in *Masc-R*/+ fat bodies, we quantified *BmVg* mRNA level by qRT-PCR. As shown in Fig 4A, the level of *BmVg* mRNA was significantly decreased in the fat body of *Masc-R*/+ females. The expression level of *BmVg* in the *Masc-R*/+ females was less than 1% compared to that of +/+ females, and was similar to that in males (Fig 4A).

Vitellogenin proteins are synthesized in fat body and released into the hemolymph during larval-pupal ecdysis [30,31]. To investigate the vitellogenin level in hemolymph of the *Masc-R*/+ females, SDS-PAGE analysis of hemolymph was performed. A protein of approximately 203 kDa, which corresponds to the molecular weight of BmVg heavy chain (BmVg-h) [31], was specifically observed in the hemolymph of +/+ females (Fig 4B, lane 1). Notably, no proteins corresponding to BmVg-h were detected by the same SDS-PAGE in the hemolymph of *Masc-R*/+ females (Fig 4B, lane 2). A protein band of approximately 42 kDa, which corresponds to the molecular weight of BmVg light chain (BmVg-l) was also very faint in the hemolymph of *Masc-R*/+ females (Fig 4B, lane 2). The protein profile displayed by the hemolymph of *Masc-R*/+ females was similar to that of males (Fig 4B; compare lane 2 with lane 3). These results suggested that the fat body in the *Masc-R*/+ female was physiologically masculinized.

### Expression of *Masc-R* caused morphological abnormalities in the ovary

**Fig 5:**
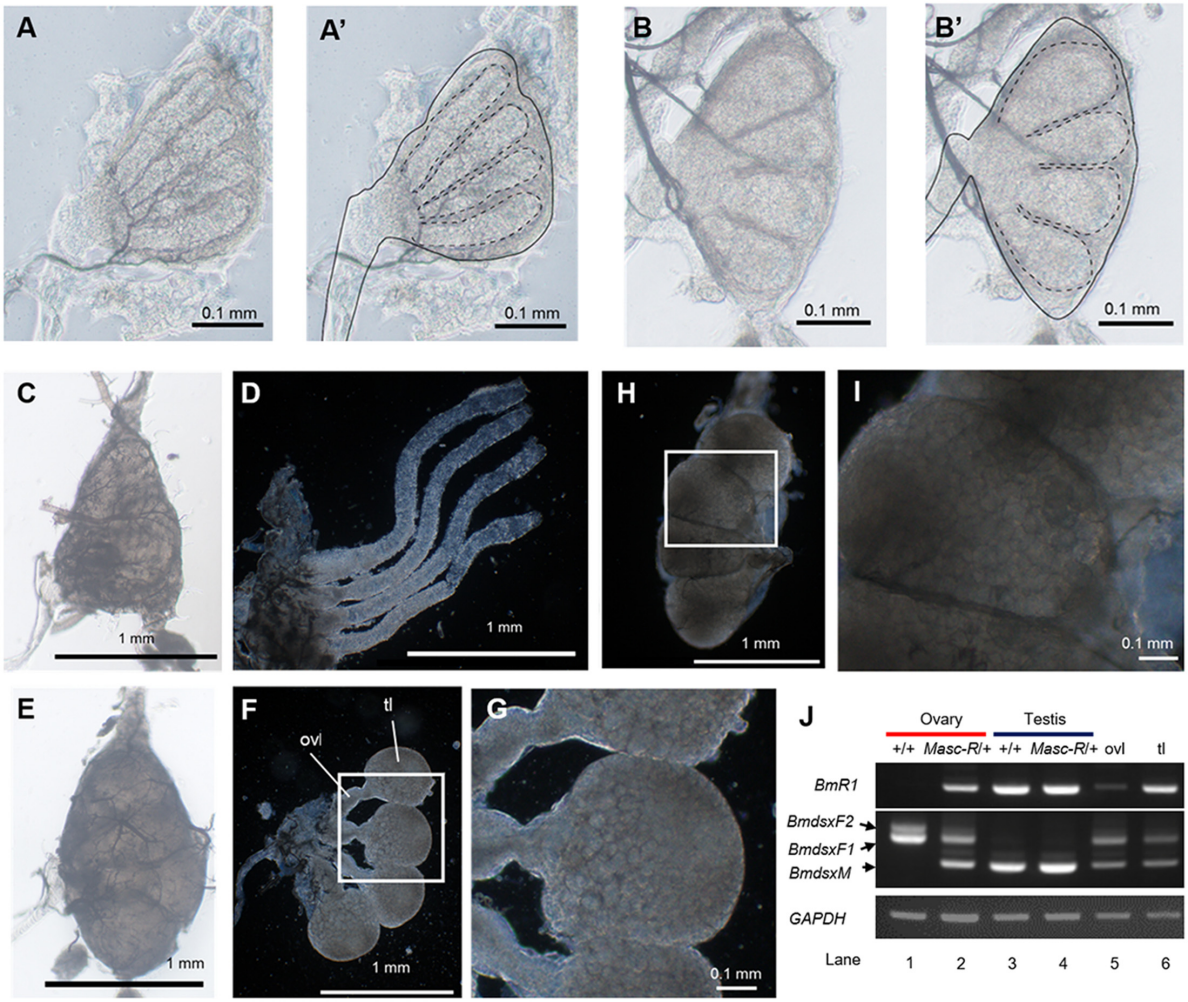
Morphological abnormalities observed in the ovary of the Sumi13-3 females. (A) (B) Ovaries dissected from the newly exuviated third instar larvae. (A) Sumi13-3 +/+ female; (B) Sumi13-3 *Masc-R*/+ female. (A’) (B’) The dotted lines indicate ovarioles. The solid line indicates basement membrane. (C) (E) The ovaries of the newly exuviated fifth instar larvae of Sumi13-3 +/+ females (C) and *Masc-R*/+ females (E). (D) The basement membrane of the ovary in C was removed. (F) The basement membrane of the ovary in E was removed. (G) The boxed area in F is enlarged. (H) The testis of the newly exuviated +/+ male fifth instar larvae. The basement membrane was removed. (I) The boxed area in H is enlarged. (J) *BmR1* and *Bmdsx* mRNA expression patterns of the gonads, which were collected from newly exuviated fifth instar larvae, were analyzed by RT-PCR. The same RT-PCR was performed with cDNAs prepared from either the *Masc-R*/+ ovaries without the globular tissues (lane 5) or the globular tissues alone (lane 6). Amplified products were separated by 1% agarose gel electrophoresis. The top panel shows *BmR1* expression. The middle panel indicates the female- and male-specific splice variants of *Bmdsx* (*BmdsxF1*, *BmdsxF2*, and *BmdsxM*, respectively). The bottom panel shows amplification of the *GAPDH* transcript, which served as a positive control for RNA extraction and RT-PCR. ovl: ovariole, tl: testis-like globular tissue.

To investigate the effects of *Masc-R* expression on female development, we next performed morphological analysis using a stereoscopic microscope. The results showed that the ovaries of *Masc-R*/+ females had morphological abnormalities at the third instar larval stage. The ovarioles of the +/+ females at the same stage became longer and took on a tubular form (Fig 5A and 5A’). On the other hand, the ovarioles in the *Masc-R*/+ females were hypertrophied and much wider than those in the +/+ females (Fig 5B and 5B’). RT-PCR analysis verified that *Masc-R* was expressed in the ovaries of *Masc-R*/+ females (S3B Fig). Consistent with this, both *BmdsxM* and *Imp*^*M*^ were obviously expressed in *Masc-R*/+ ovaries (S3B Fig).

**Fig 6:**
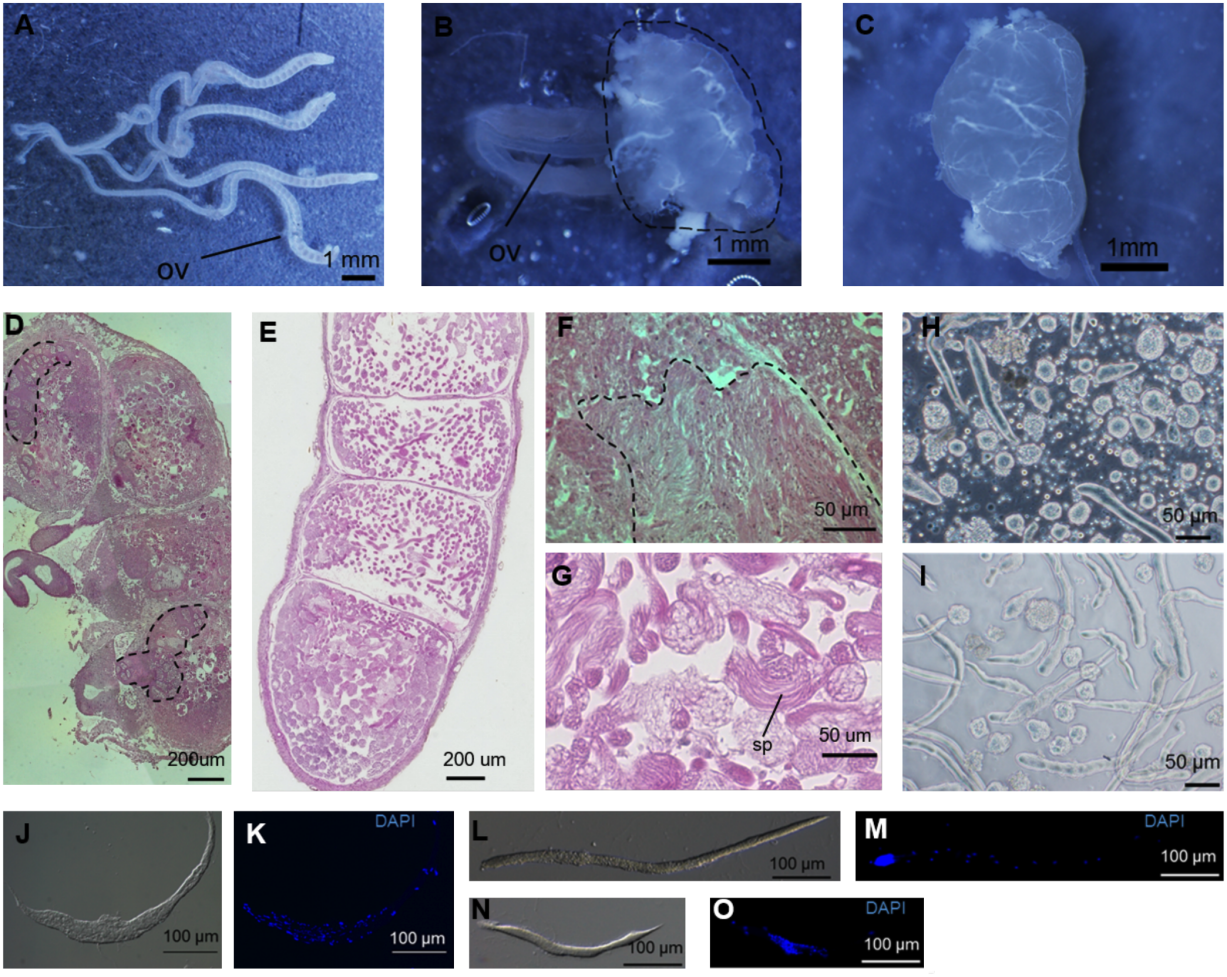
Sperm bundles were produced in the globular tissues of Sumi13-3. (A) (B) The ovaries from the day 0 Sumi13-3 +/+ female pupa (A) or the *Masc-R*/+ female pupa at the same stage (B). Dotted line in (B) indicates the globular tissues. ov, oviduct. (C) The testis from the day 0 +/+ male pupa. (D) Mid-sagittal section of the globular tissue was subjected to HE staining. The structure similar to the ovariole is surrounded by a dotted line. (E) Mid-sagittal section of the testis at the +/+ male fifth instar larval stage was subjected to HE staining. (F) Magnified view of the mid-sagittal section in (D). Fibrous structures, which likely correspond to the sperm bundles, are surrounded by a dotted line. (G) Magnified view of the (E). sp, sperm bundle. (H) The contents isolated from the abnormal tissues were observed with a phase-contrast microscope. (I) The sperm bundles in the testis of the +/+ male fifth instar larva were observed with a phase-contrast microscope. (J) (K) The sperm bundles in the *Masc-R*/+ ovary were stained with DAPI. The transmission image (J) and the fluorescent image (K) are shown. (L) (M) The eupyrene sperm bundle in the testis of the +/+ male fifth instar larva was stained with DAPI. Nuclei were condensed at the anterior pole of the bundle. The transmission image (L) and fluorescent image (M) are shown. (N) (O) The elongation stage of the apyrene sperm bundle in the testis of the +/+ male fifth instar larva was stained with DAPI. In apyrene bundles, nuclei and cytoplasmic debris were eliminated by squeezing. The transmission image (N) and fluorescent image (O) are shown.

Ovarioles in +/+ females became much longer at the fifth instar larval stage (Fig 5C and 5D), while elongation of each ovariole was not observed in the *Masc-R*/+ females. Instead, a large globular tissue was developed at the apical end of each ovariole (Fig 5E and 5F). The globular tissues were filled with small follicles (Fig 5G). These follicles showed the appearance of cysts, which were also observed in the testis at the same stage (Fig 5H and 5I). Immediately after pupation, ovarioles of the +/+ females showed intensive growing, and each contained a large number of growing eggs (Fig 6A). In the *Masc-R*/+ females, the length of each ovariole was greatly shorter than the +/+ females (Fig 6B). The globular tissues observed in Fig 5F were fused and formed an undefined tissue at the apical end of each ovariole (Fig 6B). To examine whether the *Masc-R*/+ ovaries contain tissues partially developed into testis, we performed RT-PCR analysis using primers that anneal to the *B*. *mori* homolog of *radial spoke head protein 1* gene (*BmR1*), which is specifically expressed in the testis [32]. RT-PCR detected an approximately 850 bp of *BmRI* cDNA from +/+ and *Masc-R*/+ testes but not from +/+ ovary (Fig 5J, compare lane 1 with lanes 3 and 4). Thus, our RT-PCR analysis confirmed the testis-specific expression of *BmR1* as reported previously [32]. The RT-PCR analysis also showed that *BmR1* was expressed not only in testes, but also in the *Masc-R*/+ ovaries of the fifth instar larvae (Fig 5J, lanes 2 and 4). To further confirm that the globular tissues observed in *Masc-R*/+ ovaries express *BmR1*, we performed RT-PCR with cDNAs prepared from the globular tissues and the other parts of the *Masc-R*/+ ovary. The results suggested that the expression of *BmR1* in globular tissues seemed higher than that in the other parts (Fig 5J, lane 6). These results indicated that expression of *Masc-R* facilitates partial testis developments.

### Expression of *Masc-R* in the ovary induces the formation of sperm bundles

To further verify the structures of the abnormal tissues observed in the *Masc-R*/+ females, we prepared tissue sections of the abnormal tissues. The results showed that the tissue consisted of four chambers whose shape was very similar to that of the testicular follicle (Fig 6D and 6E). A structure that seemed to be the ovariole was observed in the chambers (Fig 6D, surrounded by a dotted line). Notably, fibrous structures, which likely correspond to the sperm bundles, were also observed (compare Fig 6F with Fig 6G). Examination of contents found within the abnormal tissues using a phase-contrast microscope revealed a considerable number of sperm bundles similar to those observed in the testis of the fifth instar larvae (compare Fig 6H with Fig 6I). Nuclear staining with DAPI revealed that the nuclei were sparsely present in the sperm bundle (Fig 6K). In *B*. *mori*, males produce two types of sperm bundles, one of which consists of eupyrene sperms (Fig 6L and 6M) and the other of which is composed of apyrene sperms (Fig 6N and 6O). The patterns observed with DAPI staining showed that the sperm bundles in abnormal tissues likely corresponded to the apyrene sperm bundles. These results suggested that formation of sperm bundles was induced in the ovary by ectopic expression of *Masc-R*.

### Expression of *Masc-R* caused partial maleness in the external structures of the adult female

**Fig 7:**
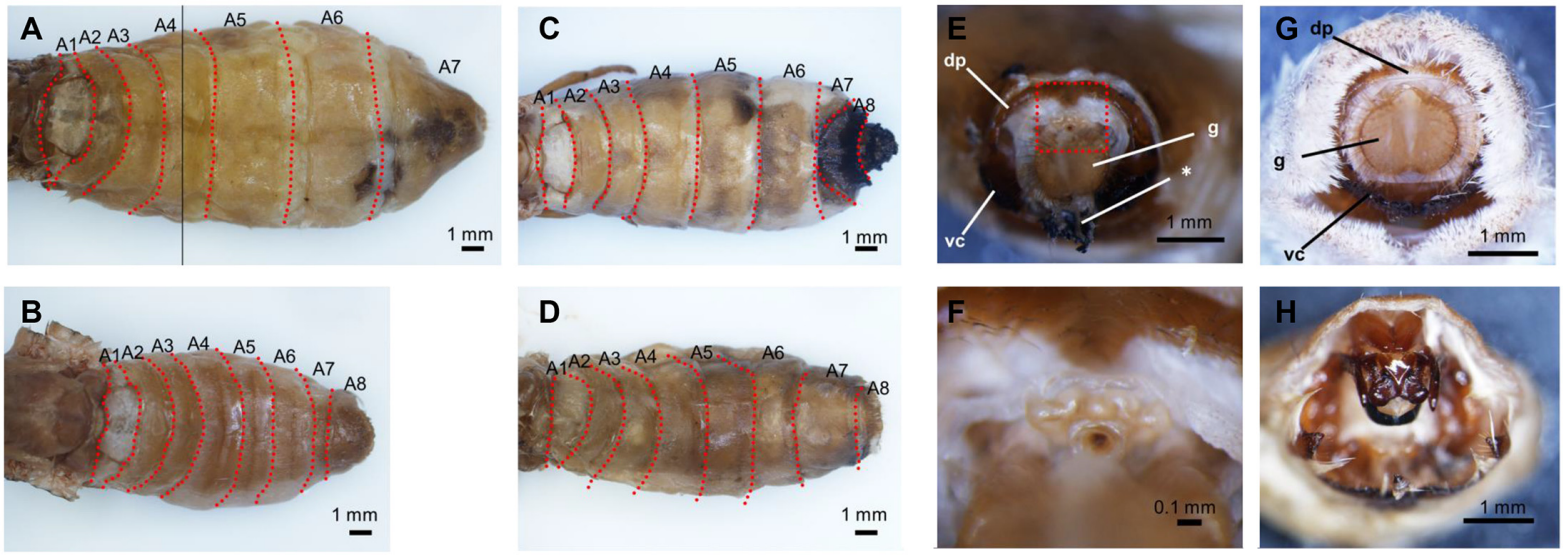
External structures of the adult Sumi13-3 females homozygous for the *Masc-R* transgene. (A) (B) Dorsal view of the abdominal segments in the adult Sumi13-3 +/+ female (A) and the +/+ male (B). (C) (D) Dorsal view of the abdominal segments in the Sumi13-3 females homozygous for the *Masc-R* transgene (*Masc-R*/*Masc-R* females). (E) A frontal view of the external genitalia in the *Masc-R*/*Masc-R* female. (F) Magnified view of the boxed area in (E). (G)(H) Frontal views of the external genitalia in the +/+ female (G) and the +/+ male (H). dp, dorsal chitin plate; g, genital papilla; vc, ventral chitin plate; * abnormal black tissue.

We observed external structures of the *Masc-R*/+ adult females, and found that they all exhibited the normal adult female phenotype (Fig 7A). Next, we crossed *Masc-R*/+ females with *Masc-R*/+ males and observed the offspring. We found all examined females homozygous for the transgene (*Masc-R*/*Masc-R* females) (S4 Fig) had an additional abdominal segment (eighth abdominal segment, A8) (Fig 7C and 7D), which is one of the male-specific external structures in *B*. *mori* (Fig 7B). Several abnormal structures, some of which were severely melanized, were also observed around the genital papilla of *Masc-R*/*Masc-R* females (Fig 7E and 7F). One of these abnormal structures, which was observed above the genital papilla, was considered to be an uncus (Fig 7F and 7H). These results suggested that the homozygous expression of *Masc-R* caused partial maleness in the external structures of the adult female.

**Fig 8:**
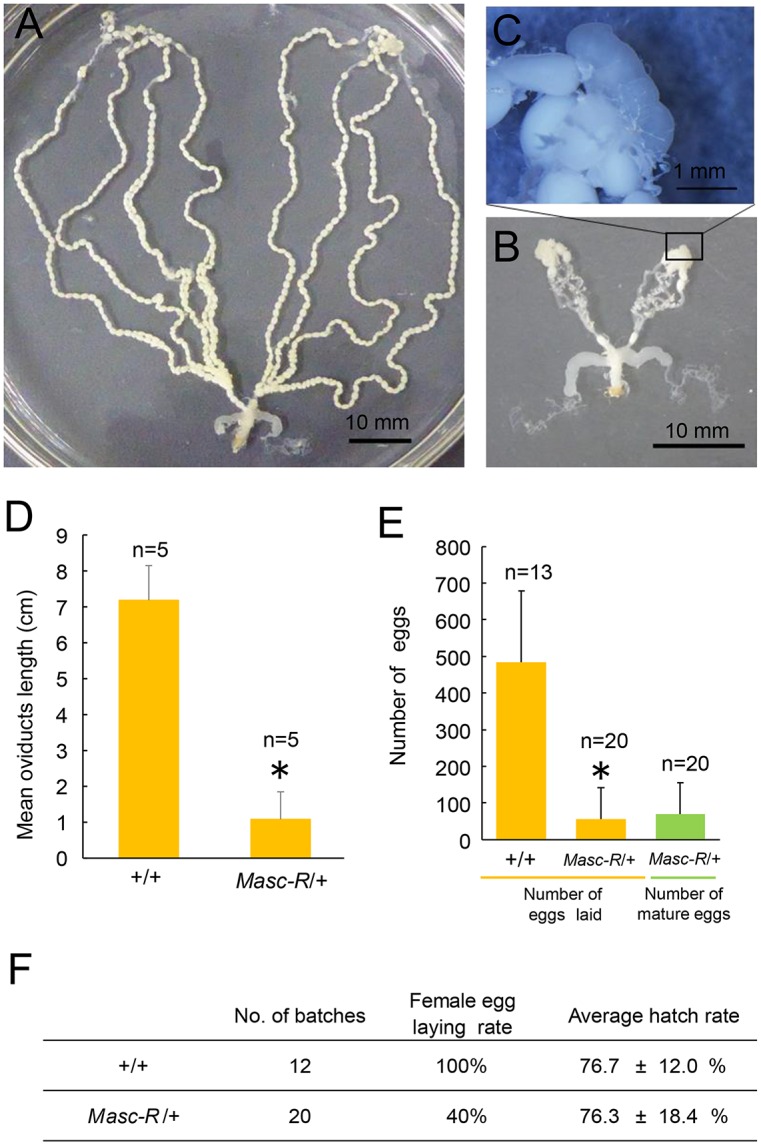
Expression of *Masc-R* reduces female fertility. (A) (B) Ovarioles of the Sumi13-3 +/+ adult female (A) and the *Masc-R*/+ female (B). (C) Magnified view of the boxed area in (B). (D) The mean length of the oviducts. Error bar: SD; * significant differences at the 0.05 level (Welch’s t-test) compared with the +/+ female. (E) Mean number of laid eggs and mature eggs remaining in the ovarioles. Error bar: SD; * significant differences at the 0.05 level (Welch’s t-test) compared with the +/+ females. (F) Hatchability of eggs laid by the +/+ females and the *Masc-R*/+ females.

We next investigated the fertility of the females expressing the *Masc-R* transgene. Ovarioles in the *Masc-R*/+ female were significantly shorter than those in the +/+ female (Fig 8A, 8B and 8D), and contained a significantly lower number of mature eggs (Fig 8E). Testis-like abnormal tissues were observed at the apical end of ovarioles (Fig 8C). The average number of eggs laid by the *Masc-R*/+ females was only 57.0, while that of the +/+ females was 483.8 (Fig 8E). Hatchability of eggs laid by the *Masc-R*/+ females was similar to that of the +/+ females (Fig 8F). These results indicated that expression of *Masc-R* severely repressed the development of ovarioles, reducing egg production.

## Discussion

In the present study, we investigated the biological functions of the *Masc* gene by transgenic approaches. Two transgenic strains, Sumi13-1 and Sumi13-3, carrying a construct with *Masc-R* under the control of UAS were generated (Fig 1). Forced expression of the UAS-*Masc-R* transgene mediated by ubiquitously expressed GAL4 caused female-specific lethality in the larval stages (Fig 2). Masc protein globally repressed gene expression from the Z chromosome to compensate for gene dosage between male (ZZ) and female (ZW) [23]. The female-specific lethality observed in this study is probably due to decreased levels of Z-linked genes in females caused by forced expression of *Masc*. This hypothesis is supported by the observation that a failure of dosage compensation is lethal during development in mice, *C*. *elegans*, and *D*. *melanogaster* [33–35]. Alternatively, high level of *Masc* expression might either repress expression of a gene(s) that is essential for female development or induce expression of a factor(s) that has a deleterious effect on normal female development. Research is in progress to further define the link between lethality and defects in dosage competition in *Masc-R* expressing females.

BmA3-GAL4-induced expression of *Masc-R* resulted in the expression of *BmdsxM* in females (S2 Fig). Although expression level of *Masc-R* in these females was much greater than that in normal males, the expression pattern of *Bmdsx* was not completely shifted from female to male mode. One plausible explanation for this discrepancy is that BmA3-GAL4 driver was not able to induce ubiquitous expression of *Masc-R*. Alternatively, other factors, in addition to *Masc*, may be necessary for fully masculinizing the expression pattern of *Bmdsx*.

We found that one of the UAS-*Masc-R* strains, Sumi13-3, expressed *Masc-R* mRNA independent of GAL4 induction in several organs (Fig 3A). Sumi13-3 females expressed both male- and female-specific splice isoforms of *Bmdsx* and *Imp*^*M*^ (Fig 3B, 3D and 3E), consistent with previous reports in which overexpression of *Masc-R* in BmN4 cells resulted in the expression of both *BmdsxM* and *Imp*^*M*^ [23,29]. However, it is possible that insertion of the UAS-*Masc*-R transgene changed the expression pattern of genes located near the insertion site, affecting the expression patterns of sex-determining genes. To rule out this possibility, we investigated expression levels of genes present within 100 kbp upstream or downstream of the *Masc-R* insertion site of Sumi13-3. There were three predicted genes (Bmgn015522, Bmgn012518, Bmgn012517) present downstream of the insertion site (Fig 1C). The qRT-PCR analysis with cDNAs prepared from day-1 first instar larvae shown in S5 Fig demonstrated that none of the three genes showed any significant differences in expression levels between Sumi13-3 females and normal females (S5 Fig). Therefore, the abnormal expression of genes near the *Masc-R* insertion site does not cause maleness in Sumi13-3 females.

In Sumi13-3, females heterozygous for the UAS-*Masc-R* transgene showed an almost complete blockade of vitellogenin synthesis (Fig 4B) and exhibited severe abnormalities in the ovaries (Fig 5B and 5F) with testis-like tissues (Fig 5G). Notably, testis-like tissues in the pupal stage contained a large number of sperm bundles (Fig 6F and 6H). In addition to these male-like phenotypes, homozygous expression of UAS-*Masc-R* resulted in formation of an additional abdominal segment, which is one of the male-specific morphological features, and caused partial male differentiation in female genitalia (Fig 7C–7E). These results demonstrated that *Masc-R* mRNA expression from the transgene led to the partial female-to male sex reversal. We hypothesized that the expression level of *Masc-R* in Sumi13-3 females was able to partially induce masculinization but was insufficient to cause lethality.

Vitellogenin is predominantly synthesized in the female fat body during larval—pupal ecdysis [30,31]. Gel-mobility shift assays demonstrate that BmDSX proteins bind to the sequence (ACATTGT) between −95 and −89 nt relative to the transcriptional initiation site of the vitellogenin gene [36]. *BmdsxF* ectopically expressed in males induces the expression of vitellogenin mRNA in male fat body [36], whereas the expression of *BmdsxM* decreases the expression level of the vitellogenin gene [37]. The TALEN (transcription activator-like effector nuclease)-based mutation of BmDSXF reduced expression of the vitellogenin mRNA, whose expression level was only 25% compared to that in the normal-type females [38]. These findings suggest that *Bmdsx* directly controls the transcriptional level of the vitellogenin gene. The dramatic reduction in vitellogenin expression observed in Sumi13-3 *Masc-R*/+ females could be attributed to the increased expression of BmDSXM caused by *Masc-R* expression.

Females homozygous for the *Masc-R* transgene exhibited abnormalities in genitalia with partial male structures. This is similar to our previous report that ectopic expression of BmDSXM in females caused partial male differentiation in female genitalia [37]. Moreover, an eighth segment was formed in these females. Sexual dimorphisms have been reported in adult abdominal segments of *D*. *melanogaster*. In this species, the female genitalia is developed from the eighth abdominal segment, while the male genitalia originated from the ninth abdominal segment [39,40]. It is also known that *Abdominal-B* (*Abd- B*) is responsible for the specification of these posterior segments [41]. In *Drosophila melanogaster*, DsxF and DsxM cooperate with Abd-B isoforms to produce sexual dimorphisms in these posterior segments [42]. In *B*. *mori*, the expression level of *Abd-B* in the posterior abdomen differs between males and females [43]. Taken together, in females homozygous for the UAS-*Masc-R* transgene, interaction between BmDSXM and Abd-B in the posterior abdomen might produce a developmental signal that facilitates the formation of the male-specific eighth segment.

In this study, the ectopic expression of *Masc-R* did not induce complete sex reversal in females. As described above, Sumi13-3 females expressed not only *BmdsxM* transcripts, but also *BmdsxF* transcripts (Fig 3B, S3 Fig). This may be due to an insufficient level of *Masc-R* expression for inducing female-to-male sex reversal because full activation of *Masc-R* will cause lethality in females during the larval stage. It has been reported that BmDSXF and BmDSXM compete with each other for a target site when both are present [36]. This competition would interfere with the masculinizing activity of the BmDSXM protein and inhibit the feminizing activity of the endogenous BmDSXF protein in *Masc-R* transgenic females. The incomplete masculinization in the Sumi13-3 females may be considered a result of such competition. Another likely explanation for the incomplete sex reversal is that the dosage of Z-linked genes may mediate sex determination and two doses are required for male development (ZZ). Several lines of evidence strongly support this hypothesis. Mapping of testis-specific full-length cDNA sequences onto chromosomes indicates that the Z chromosome is enriched in testis-specific genes [44]. The mean expression level of the Z-linked genes in testis is approximately 11 times higher than in the ovary. In addition, expression levels of 55% genes on the Z chromosome are at least two times higher in testis than in ovary [45]. It would be possible that the Z chromosome is enriched in genes essential for governing male sexual dimorphisms. In the chicken, whose sex is determined by the ZW system (similar to the silkworm), the Z dosage hypothesis for sex determination is supported based on the observation that two copies of a Z-linked gene, *DMRT1*, are required for male sex determination [46].

The most notable results in this study were the ectopic formation of testis that contained a considerable number of sperm bundles in females. Prior experiments show that ovaries of adult females with mutations in the female-specific *Bmdsx* exon induced by TALEN showed abnormalities closely resembling those observed in the present study [38]. These females have significantly shorter ovarioles containing little or no mature eggs, and abnormal tissues were observed at the apical end of ovarioles. Furthermore, our knockout analysis using somatic TALEN mutagenesis indicated that mutations in the female-specific exon of *Bmdsx* caused abnormalities in ovaries of 5th instar larvae (S6 Fig). Similar to the *Masc-R*/+ ovaries, a testis-like globular tissue was developed at the apical end of each ovariole. These findings suggest that loss of function mutation in BmDSXF may be sufficient for inducing the testis-like tissues in females. In *D*. *menalogaster*, transgenic analysis showed that female DSX protein functions as a negative regulator of male differentiation [47]. The loss of function mutation in BmDSXF may abolish such negative regulatory effect of BmDSXF on male differentiation, resulting in the formation of the testis-like tissues. Ectopic expression of *Masc-R* in females partially shifted the splicing pattern of *Bmdsx* from female to male mode (Figs 3B and 5J). This will not only increase the expression level of BmDSXM, but also reduce the expression level of BmDSXF. Such reduced level of BmDSXF may impair the negative regulatory effect of BmDSXF on male development, leading to the formation of the testis-like tissues.

Although loss of function mutation of BmDSXF resulted in the formation of the testis-like tissues (S6 Fig), it still remains unclear whether these testis-like tissues contain mature sperms. In *D*. *melanogaster*, *dsx* is not required within the female or male germ line for normal development [48,49] and that female germline cells undergo normal oogenesis when surrounded by a soma masculinized by the dominant male gain-of-function *dsx* allele, *dsxDom* [50]. Similarly, *Bmdsx* may be dispensable for sexual differentiation of germ cells. It is conceivable that *Masc-R* expression in ZW germ cells may directly promote the development of male germ cells. However, ectopic expression of *Masc-R* alone is not sufficient to induce ZW germ cells to complete spermatogenesis because sperms observed in *Masc-R* females were not fully matured (Fig 6K). One possible explanation for this is that piRNAs transcribed from *Fem* or other W-chromosomal regions may inhibit differentiation of *Masc-R*-expressing germ cells into mature sperms. In *B*. *mori*, W chromosome is a source of numerous female-specific piRNAs [51]. It could be postulated that female-specific piRNAs from W chromosome may disrupt expression patterns of genes crucial for normal spermatogenesis. Alternatively, two doses of Z-linked genes are required for germ cells to achieve spermatogenesis.

Recently, a *Masc* homolog has been identified from *Trilocha varians*, a species closely related to *B*. *mori* [52]. RNAi-mediated knockdown of the *Masc* homolog (*TvMasc*) shifted the splicing pattern of *Trilocha varians dsx* from male mode to female mode. Moreover, overexpression of *TvMasc* cDNA in BmN4 cells induced the expression of *BmdsxM* and *Imp*^*M*^. These findings suggested that the function of *Masc* is evolutionarily conserved in the sex determination pathway of *Bombycidae*. *Masc* encodes a CCCH-type zinc finger protein [23]. CCCH-type zinc finger proteins directly bind not only to DNA but also to RNA [53,54]. It will be important to identify direct target RNAs of Masc protein to explore the molecular mechanisms underlying gene dosage compensation and male development in lepidopteran insects.

## Materials and Methods

### Silkworm stock, production of transgenic silkworms, and crossing

*B*. *mori* strains were maintained on standard conditions [55]. Silkworms expressing *Masc-R* was generated by *piggyBac* element transformation. The *Masc-R* cDNA sequence was reported previously [23]. Plasmid DNA of the *piggyBac* transformation vector containing UAS-*Masc-R* was purified using a Qiagen Plasmid Midi Kit (Qiagen, Hilden, Germany). *PiggyBac*-mediated germline transformation was conducted to generate transgenic strains by the method of Tamura et al. [56] with minor modifications. Briefly, *w1-pnd* embryos were microinjected with DNAs (*piggyBac* transformation vector [0.4 μg/μL], *piggyBac* transposase mRNA [0.2 μg/μL], and helper plasmid DNA [0.1 μg/μL; pGEMe-pigORF-pA90]) in injection buffer. Hatched larvae from the injected eggs were grown to the adult stage, and the G0 individuals were crossed by sister-brother mating to obtain G1 progenies. EGFP-positive individuals were selected from G1 embryos to establish UAS-*Masc-R* strains. Ubiquitous GAL4 expressing strain, BmA3-GAL4 ([27]; strain name: 193–2), was used for crossing with the *Masc-R* strains to produce silkworms expressing these genes. Suzu19-1 was obtained from NBRP (National bioresource project)-Silkworm Center at Kyushu University and used to discriminate females from males based on DsRed expression. We crossed Sumi13-3 males heterozygous for the UAS-*Masc-R* transgene with Suzu19-1 females. We selected the progenies that expressed DsRed and EGFP. The progenies were heterozygous for the UAS-*Masc-R* transgene females (hereafter described as *Masc-R*/+ females). To obtain *Masc-R*/*Masc-R* females, we reared *Masc-R*/+ females by feeding fresh mulberry leaves harvested during early summer season to help *Masc-R*/+ females grow well. Then more than 100 females were crossed with *Masc-R*/+ males, yielding progenies, approximately 300 of which reached on adult stage. We selected *Masc-R*/*Masc-R* females from these adults by PCR-based genotyping as described in S4 Fig. It was extremely difficult to obtain sufficient numbers of *Masc-R*/*Masc-R* females, and therefore, we were not able to use *Masc-R*/*Masc-R* females for all the analyses. In this study, *Masc-R*/+ females were mainly subjected to the analyses.

### Histological examination

To prepare the tissue sections, tissues were fixed with 4% paraformaldehyde or Bouin's solution followed by dehydration in a graded ethanol-n-butanol series, and then embedded in paraffin. Cut sections (5 μm thick) were deparaffinized and stained with Mayer's hematoxylin-eosin (HE). Sperm bundles were placed on MAS-coated glass slides (Matsunami Glass Ind., Ltd., Osaka, Japan), air-dried and baked at 55°C for 5 min. For nuclear staining, they were covered and mounted with Vectashield containing DAPI (Vector Laboratories, Peterborough, UK).

### Plasmid construction

Transformation vector, pBacMCS [UAS-Masc-R-SV40, 3×P3-GFP], was produced by the following procedure. *Masc-R* cDNA was PCR-amplified from pIZ/V5-Masc-R-His [23] using KOD plus DNA polymerase (Toyobo Co. Ltd., Ohtsu, Japan). The following primer set was used: 5’- GCC TAG TAG ACC TAG CCA AAA TGG ATT ACA AGG ATG ACG ACG-3’, 5’- GTA TGG CTG ACC TAG CTA TTG AAA CGG CGG TGG TG-3’. *Masc-R* fragment was inserted into the *Bln*I site of the pBacMCS [UAS-SV40, 3xP3-GFP T to H] vector [55] using In-Fusion HD Cloning Plus (TaKaRa Bio Inc., Shiga, Japan).

### Southern blotting and SDS-polyacrylamide gel electrophoresis

In the Southern blot analysis, genomic DNA was isolated from adults using the method described by Sambrook and Russell [57]. Genomic DNA was digested with either *Bam*HI or *Bgl*II. Digested genomic DNAs were then separated on a 1.0% agarose gel and subsequently transferred to a Hybond-N+ membrane (GE Healthcare UK Ltd., Buckinghamshire, UK). Southern blot analysis was performed using probes labeled with Amersham AlkPhos Direct Labeling Reagents, and DNA bands were visualized using Amersham CDP-Star Detection Reagent following the manufacturer’s guidelines (GE Healthcare). SDS-PAGE analysis of hemolymph was performed according to the method of Mine et al. [30]. Hemolymph (1 μL) was treated with SDS sample buffer for 2 min at 95°C. Samples were separated by electrophoresis on 10% SDS-polyacrylamide gel and stained with Quick-CBB PLUS (Wako, Osaka, Japan).

### RNA extraction and Reverse Transcription (RT)-PCR

Total RNA was extracted from each egg using Isogen (Nippon Gene, Tokyo, Japan), as described previously [19]. RT-PCR reactions were performed according to the protocol described previously [19]. The primer sequences and PCR conditions utilized in this study are indicated in S1 Table.

### Quantitative Real-Time RT-PCR (qRT-PCR)

qRT-PCR assays were performed according to the protocol described previously [19]. All primer sequences used in this study are listed in S2 Table. The BmEF-2F1 and BmEF-2R1 primers were used to amplify elongation factor-2 (*EF-2)* as an internal standard for quantification [58].

### Genomic PCR

Genomic PCR was performed with EmeraldAmp PCR Master Mix (TaKaRa) according to the protocol described previously [59]. The primer sequences and PCR conditions utilized in this study are indicated in S1 Table.

### Inverse polymerase chain reaction (Inverse PCR)

Inverse PCR was performed as described previously [27][55]. Briefly, genomic DNA was isolated from adult legs using a DNeasy Blood & Tissue kit (Qiagen). DNA was digested with either *Msp*I or *Sau3*AI, and then circularized with T4 DNA ligase (New England Biolabs, Beverly, MA, USA) at 4°C overnight. Circularized DNA was used as a template for the nested PCR method. PCR was performed in a total reaction volume of 30 μL using TaKaRa Ex Taq (TaKaRa). In the 2^nd^ PCR, 0.5 μL of the first PCR product was used as a template.

## Supporting Information

S1 Table Primer sequences and PCR conditions utilized in this study. (DOCX)

S2 Table Sequences of primers used for qRT-PCR. (DOCX)

S1 Fig Expression of the DsRed and EGFP genes at the embryonic stage. R/+ (DsRed-positive); G/+ (EGFP-positive) animals possessed both BmA3-GAL4 and UAS-*Masc-R* transgenes. R/+ and G/+ animals carried either BmA3-GAL4 or UAS-*Masc-R*, respectively. +/+ animals had no transgenes.
(TIF)

S2 Fig Expression patterns of *Bmdsx* and expression level of *Masc-R* in *Masc-R*-expressing females. (A) Expression patterns of *Bmdsx* were analyzed by RT-PCR. Amplified products were separated by 1% agarose gel electrophoresis. The panel indicates the female- and male-specific splice variants of *Bmdsx* (*BmdsxF1* and *BmdsxM*, respectively). cDNAs prepared from larvae at L1D1. (B) Quantification of *Masc* mRNA at L1D1 using qRT-PCR. *EF2* was served as an internal standard. Error bar: SD. R/G animals possessed both BmA3-GAL4 and UAS-*Masc-R*. G/+ animals carried UAS-*Masc-R* transgene.
(TIF)

S3 Fig Expression patterns of *Masc*-*R*, *Bmdsx*, and *Imp*^*M*^ were analyzed by RT-PCR. Amplified products were separated by 1% agarose gel electrophoresis. The top panel indicates the *Masc-R* expression. The second panel from the top indicates the female- and male-specific splice variants of *Bmdsx* (*BmdsxF1*, *BmdsxF2*, and *BmdsxM*, respectively). The third panel shows *Imp*^*M*^ expression. The bottom panel shows amplification of the *GAPDH* transcript, which served as a positive control for RNA extraction and RT-PCR. *Masc-R*/+, Sumi13-3 females heterozygous for the UAS-*Masc-R* transgene; +/+, Sumi13-3 sister females, which did not have the UAS-*Masc-R* transgene. cDNAs prepared from fat bodies within 3 hours after puation (A) and ovaries at the third instar larval stage (B) were subjected to the RT-PCR analyses.
(TIF)

S4 Fig PCR-based genotyping of the Sumi13-3 females. (A) Schematic diagram of the primer locations used in the PCR-based genotyping. Red arrows indicate primers. (B) PCR products were separated by 1% agarose gel electrophoresis. The upper panel (TG-) indicates amplified products with Sumi13-3F and Sumi13-3R primers that specifically annealed to the region flanking the insertion site of the UAS-*Masc-R* transgene. The lower panel (TG+) shows the PCR amplifications using Sumi13-3F and ks129, which can amplify the DNA fragment between the transgene and its flanking genomic region. Only TG- DNA fragment was amplified from the Sumi13-3 females, which did not have the UAS-*Masc-R* transgene (+/+), while both TG- and TG+ DNA fragments were amplified from the Sumi13-3 females heterozygous for the UAS-*Masc-R* transgene (*Masc-R*/+). The same PCR reactions detected only a TG+ DNA fragment from females numbered 1 through 17. These individuals served as females homozygous for UAS-*Masc-R* transgene (*Masc-R*/*Masc-R*) in the present study.
(TIF)

S5 Fig Quantification of expression levels of Bmgn015522, Bmgn012518 and Bmgn012517 genes. qRT-PCR was performed to quantify mRNA levels of Bmgn015522, Bmgn012518, and Bmgn012517 genes at L1D1. *EF2* was used as an internal standard. Error bar: SD.
(TIF)

S6 Fig Morphological abnormalities observed in the ovary of females with mutations of *BmdsxF* by somatic TALEN mutagenesis. Knockout silkworms were generated using transcription activator-like effector nuclease (TALENs), as described previously [60, 61]. mRNAs encoding a transcription activator-like effector nuclease (TALEN) that targeted *Bmdsx* were injected into eggs, and hatched larvae (G0 animals) were subjected to the phenotypic analysis. (A) Target site of TALENs within female specific exon of *Bmdsx*. Splicing patterns of *Bmdsx* gene from male and female are shown. Exons are described by box, and female specific exons are in black. TAL effector-binding sequences within the female-specific exon 3 are shown in blue. (B) Variation of sequences around the TALEN target site from two G0 female lines (No. 5 and 6). Molecular sexing of G0 animals was determined by PCR using W chromosome RAPD markers, *Musashi*. The native sequence is shown at the top of the alignment (Bmdsx). Deleted nucleotides are shown as dashed line in red. 6–5 contained unknown inserted sequences (red) and lacked approximately 1 kbp of sequence downstream of the target site. The ovaries of the day-5 fifth instar larvae of normal females (C) and G0 females with mutations in *BmdsxF* (D). (E) The abnormal globular tissue observed in the ovary of the day-12 G0 female pupa.
(TIF)
