# Supplementary material for: Transgenic Expression of the piRNA-Resistant Masculinizer Gene Induces Female-Specific Lethality and Partial Female-to-Male Sex Reversal in the Silkworm, Bombyx mori
Source: PLoS Genet. 2016 Aug 31;12(8):e1006203. doi: 10.1371/journal.pgen.1006203 (PMC5007099; doi:10.1371/journal.pgen.1006203)
Supplement: S1 Table — (DOCX) [file pgen.1006203.s001.docx]

S1 Table1 Primer sequences and PCR conditions utilized in this study

|  |  |  |  |  |  |  | |  |  |  |  |  |  |  |  |  |
| --- | --- | --- | --- | --- | --- | --- | --- | --- | --- | --- | --- | --- | --- | --- | --- | --- |
|  |  |  |  | Primers |  | Sequence | |  | Denaturation |  | Annealing |  | Elongation |  | N°**c**ycles |  |
|  |  | *Masc-R* |  | MascRF1 |  | ATGGATTACAAGGATGACGAC | |  | 98℃ |  | 57℃ |  | 72℃ |  | 30 |  |
|  |  |  |  | MascRR1 |  | CCAGAGGAAGTTGCGACAAAG | |  | 10 s |  | 30 s |  | 60 s |  |  |  |
|  |  | *Bmdsx* |  | FDSX-F2 |  | CGCCTTACCGCAGACAGGCAG (Sakai et al.,2014) | |  | 98℃ |  | 57℃ |  | 72℃ |  | 35 |  |
|  |  |  |  | FDSX-R4 |  | GCGCAGTGTCGTCGCTACAAGG (Sakai et al., 2014) | |  | 10 s |  | 30 s |  | 60 s |  |  |  |
|  |  | *Imp^M^* |  | BmIMP-F |  | ATGGACGGTGACATGTCTCAAG (Suzuki et., 2010) | |  | 98℃ |  | 55℃ |  | 72℃ |  | 30 |  |
|  |  |  |  | BmIMP-R |  | TCATCCCGCCTCAGACGATTG (Sakai et al.,2014) | |  | 10 s |  | 30 s |  | 60 s |  |  |  |
|  |  | *BmR1* |  | BmR1F |  | GCTGGGCTGTTCTACCGAAT(Xu et al., 2015) | |  | 98℃ |  | 55℃ |  | 72℃ |  | 35 |  |
|  |  |  |  | BmR1R |  | TCCCAACATCTAATCCTTCTG | |  | 10 s |  | 30 s |  | 60 s |  |  |  |
|  |  | *GAPDH* |  | GAPDH-F |  | CATGAACAGTAGTCATCAAGC (Sakai et al., 2014) | |  | 98℃ |  | 55℃ |  | 72℃ |  | 26 |  |
|  |  |  |  | GAPDH-R |  | GCCGCATTGGCCGTTTGGTGC (Sakai et al., 2014) | |  | 10 s |  | 30 s |  | 60 s |  |  |  |
|  |  | TG- |  | Sumi13-3F |  | TGCCGATGTTTATGAAGAACGGTAGTC | |  | 98℃ |  | 57℃ |  | 72℃ |  | 30 |  |
|  |  |  |  | Sumi13-3R |  | CCTTAATCGTGGAAGTGATTCGTG | |  | 10 s |  | 30 s |  | 60 s |  |  |  |
|  |  | TG+ |  | Sumi13-3F |  | TGCCGATGTTTATGAAGAACGGTAGTC | |  | 98℃ |  | 57℃ |  | 72℃ |  | 30 |  |
|  |  |  |  | ks129 |  | AAATCAGTGACACTTACCGCATT | |  | 10 s |  | 30 s |  | 60 s |  |  |  |
|  |  |  |  |  |  | |  |  |  |  |  |  |  |  |  |  |
