# Supplementary material for: Transgenic Expression of the piRNA-Resistant Masculinizer Gene Induces Female-Specific Lethality and Partial Female-to-Male Sex Reversal in the Silkworm, Bombyx mori
Source: PLoS Genet. 2016 Aug 31;12(8):e1006203. doi: 10.1371/journal.pgen.1006203 (PMC5007099; doi:10.1371/journal.pgen.1006203)
Supplement: S2 Table — (DOCX) [file pgen.1006203.s002.docx]

S2 Table Sequences of primers used for qRT-PCR

|  |  | Gene |  | Primers |  | Sequence |  |
| --- | --- | --- | --- | --- | --- | --- | --- |
|  |  | *BmVg* |  | BmVgF |  | CAGCGGAAGTGCTTTCAAAGC |  |
|  |  |  |  | BmVgR |  | TTGAGATCAACAGGCAGTTCC |  |
|  |  | *Bmgn 012518* |  | 012518F |  | GTGTTTGTCTCGCATCATTCC |  |
|  |  |  |  | 012518R |  | AATTGCATGCCGCAAACGTC |  |
|  |  | *Bmgn 012517* |  | 012517F |  | TGACTATCTCGGAGATCTTTG |  |
|  |  |  |  | 012517R |  | GACGACTTTAAACGCAGCCG |  |
|  |  | *Bmgn 015522* |  | 015522F |  | TCCACAACGATCCACTTATTG |  |
|  |  |  |  | 015522R |  | TCTCAGGGGTCCTAATGGAG |  |
|  |  | *Fem* |  | FemQ-F |  | TGGTTTATACAATCGATCCACTG (Sakai et al., 2015) |  |
|  |  |  |  | FemQ-R |  | CTGACTCACTTAAATTCGATCTC (Sakai et al., 2015) |  |
|  |  | *Masc* |  | MascF |  | GCTAAAATTGCTGGGATTGCTA (Kiuchi et al., 2014) |  |
|  |  |  |  | MascR |  | CAGCAATCGGAATTTTCTTCTG (Kiuchi et al., 2014) |  |
|  |  | *Imp^M^* |  | BmIMPE7-F |  | ATGCGGGAAGAAGGTTTTATG (Sakai et al.,2014) |  |
|  |  |  |  | BmIMP-R |  | TCATCCCGCCTCAGACGATTG (Sakai et al.,2014) |  |
