# Supplementary figures and images for: Transgenic Expression of the piRNA-Resistant Masculinizer Gene Induces Female-Specific Lethality and Partial Female-to-Male Sex Reversal in the Silkworm, Bombyx mori
Source: PLoS Genet. 2016 Aug 31;12(8):e1006203. doi: 10.1371/journal.pgen.1006203 (PMC5007099; doi:10.1371/journal.pgen.1006203)

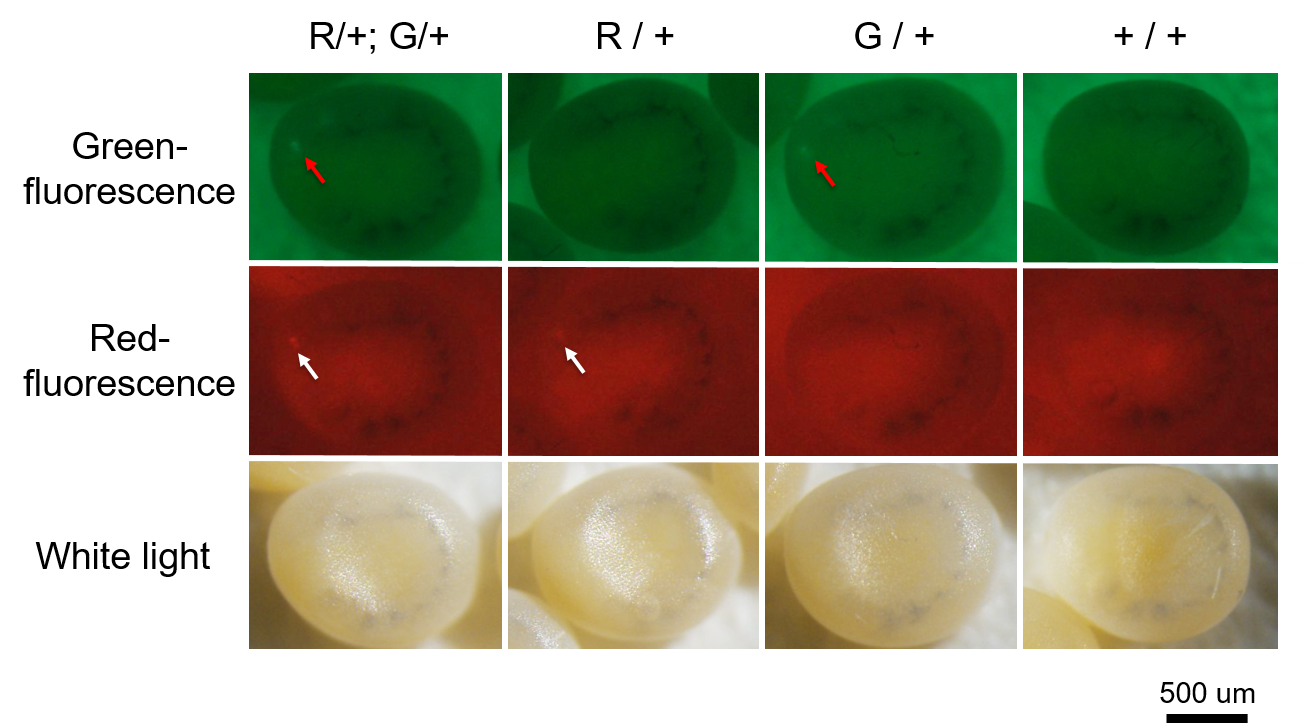

Supplement: S1 Fig — R/+ (DsRed-positive); G/+ (EGFP-positive) animals possessed both BmA3-GAL4 and UAS-Masc-R transgenes. R/+ and G/+ animals carried either BmA3-GAL4 or UAS-Masc-R, respectively. +/+ animals had no transgenes. (TIF) [file pgen.1006203.s003.tif]

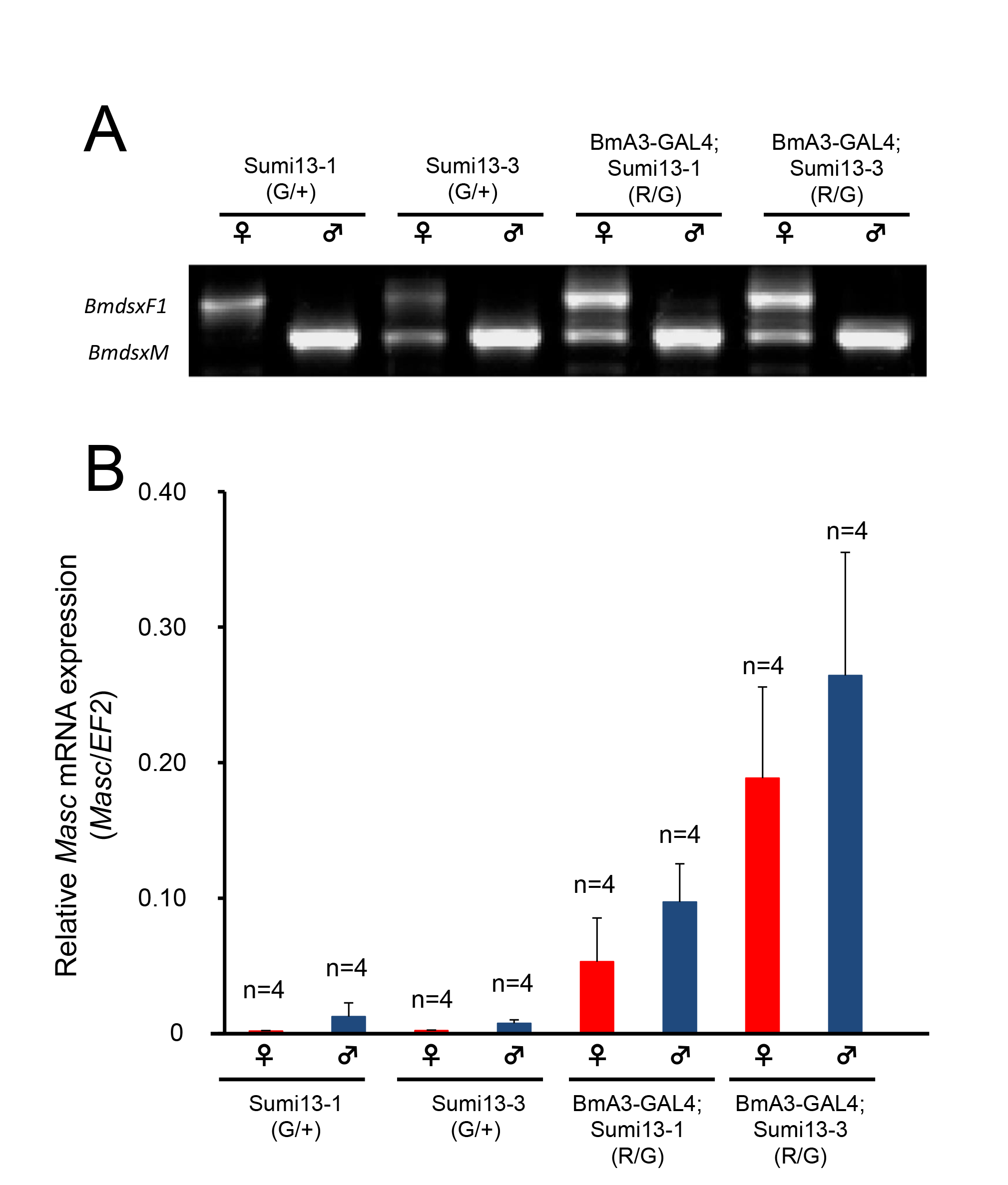

Supplement: S2 Fig — (A) Expression patterns of Bmdsx were analyzed by RT-PCR. Amplified products were separated by 1% agarose gel electrophoresis. The panel indicates the female- and male-specific splice variants of Bmdsx (BmdsxF1 and BmdsxM, respectively). cDNAs prepared from larvae at L1D1. (B) Quantification of Masc mRNA at L1D1 using qRT-PCR. EF2 was served as an internal standard. Error bar: SD. R/G animals possessed both BmA3-GAL4 and UAS-Masc-R. G/+ animals carried UAS-Masc-R transgene. (TIF) [file pgen.1006203.s004.tif]

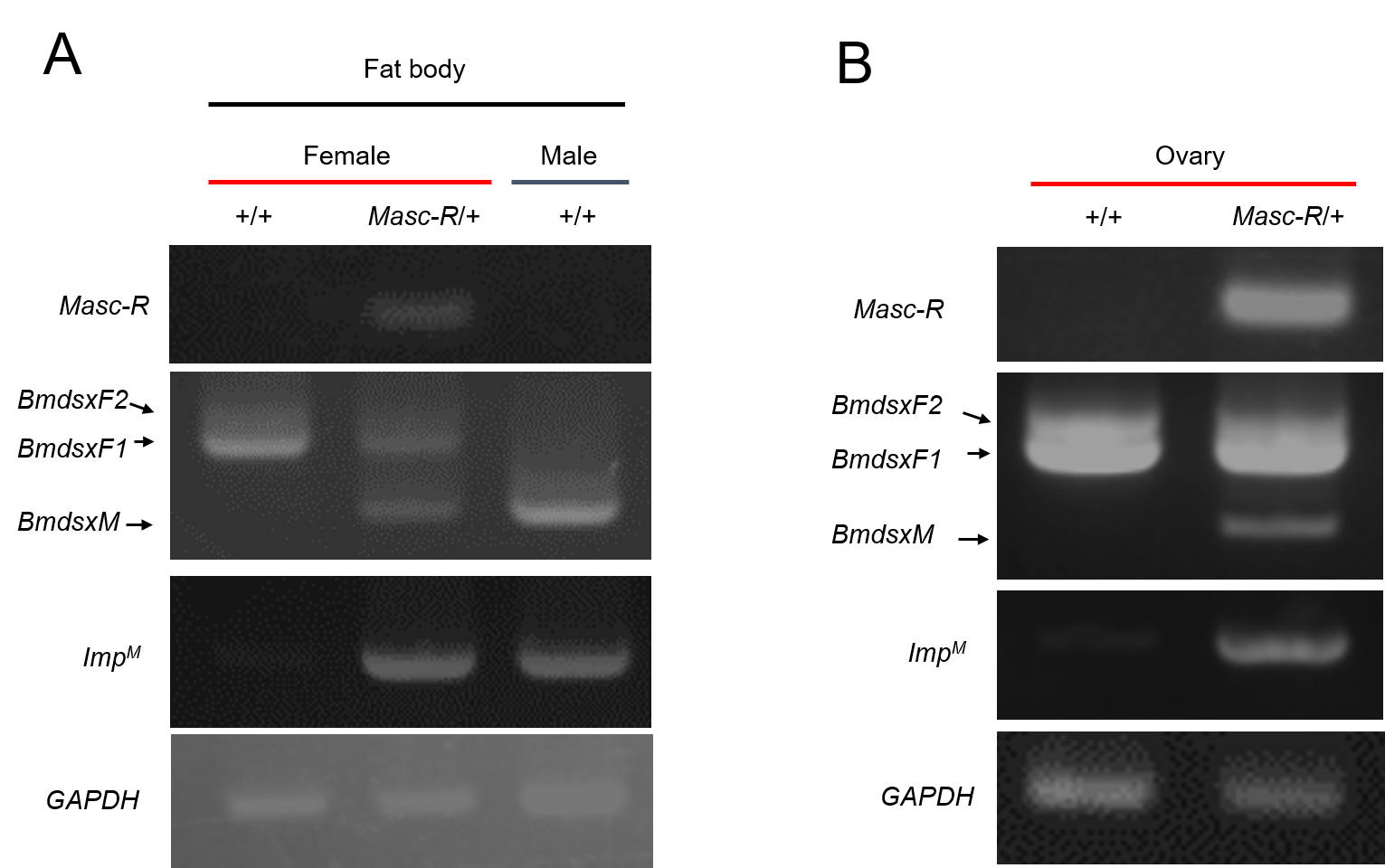

Supplement: S3 Fig — Amplified products were separated by 1% agarose gel electrophoresis. The top panel indicates the Masc-R expression. The second panel from the top indicates the female- and male-specific splice variants of Bmdsx (BmdsxF1, BmdsxF2, and BmdsxM, respectively). The third panel shows ImpM expression. The bottom panel shows amplification of the GAPDH transcript, which served as a positive control for RNA extraction and RT-PCR. Masc-R/+, Sumi13-3 females heterozygous for the UAS-Masc-R transgene; +/+, Sumi13-3 sister females, which did not have the UAS-Masc-R transgene. cDNAs prepared from fat bodies within 3 hours after puation (A) and ovaries at the third instar larval stage (B) were subjected to the RT-PCR analyses. (TIF) [file pgen.1006203.s005.tif]

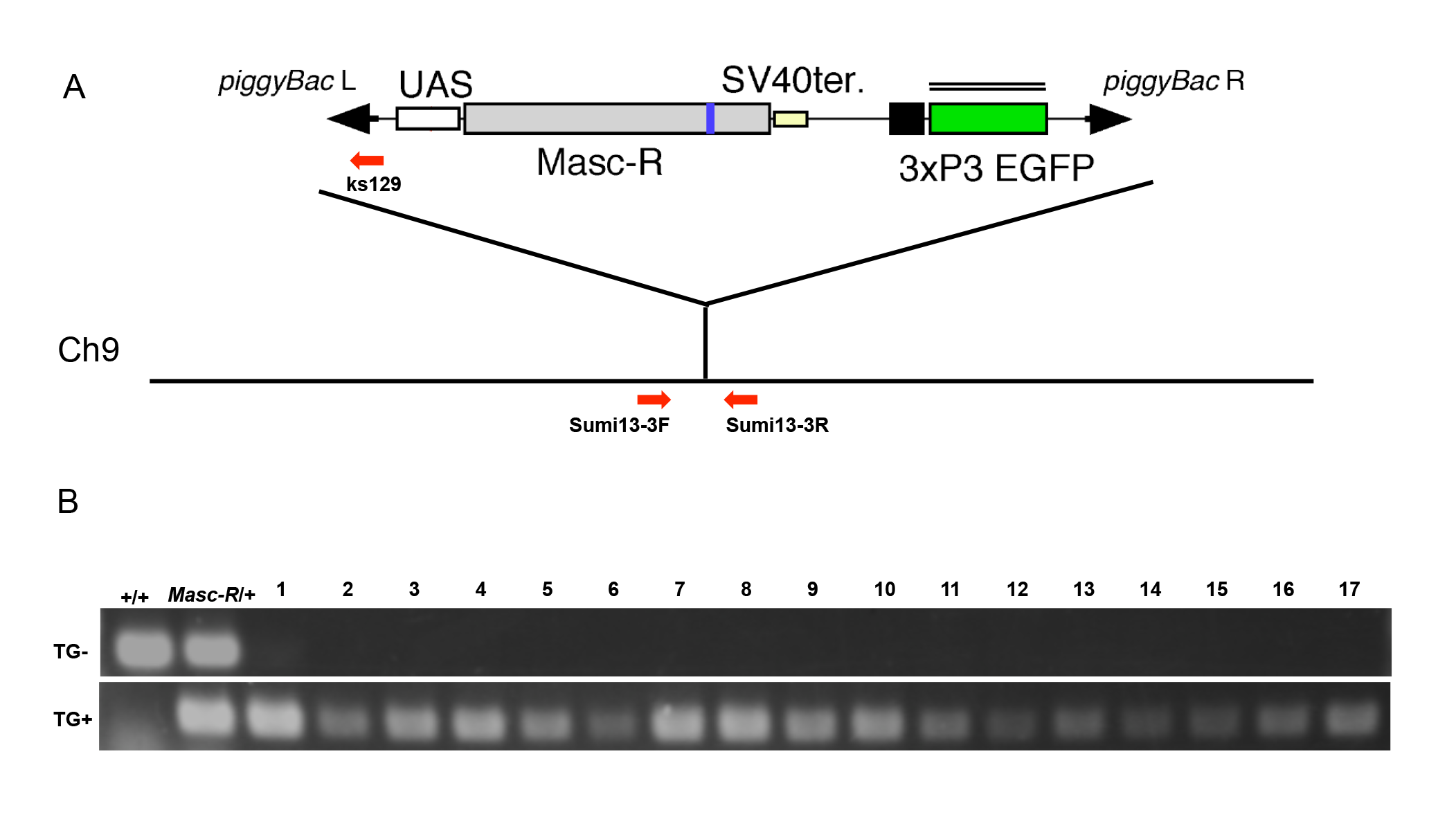

Supplement: S4 Fig — (A) Schematic diagram of the primer locations used in the PCR-based genotyping. Red arrows indicate primers. (B) PCR products were separated by 1% agarose gel electrophoresis. The upper panel (TG-) indicates amplified products with Sumi13-3F and Sumi13-3R primers that specifically annealed to the region flanking the insertion site of the UAS-Masc-R transgene. The lower panel (TG+) shows the PCR amplifications using Sumi13-3F and ks129, which can amplify the DNA fragment between the transgene and its flanking genomic region. Only TG- DNA fragment was amplified from the Sumi13-3 females, which did not have the UAS-Masc-R transgene (+/+), while both TG- and TG+ DNA fragments were amplified from the Sumi13-3 females heterozygous for the UAS-Masc-R transgene (Masc-R/+). The same PCR reactions detected only a TG+ DNA fragment from females numbered 1 through 17. These individuals served as females homozygous for UAS-Masc-R transgene (Masc-R/Masc-R) in the present study. (TIF) [file pgen.1006203.s006.tif]

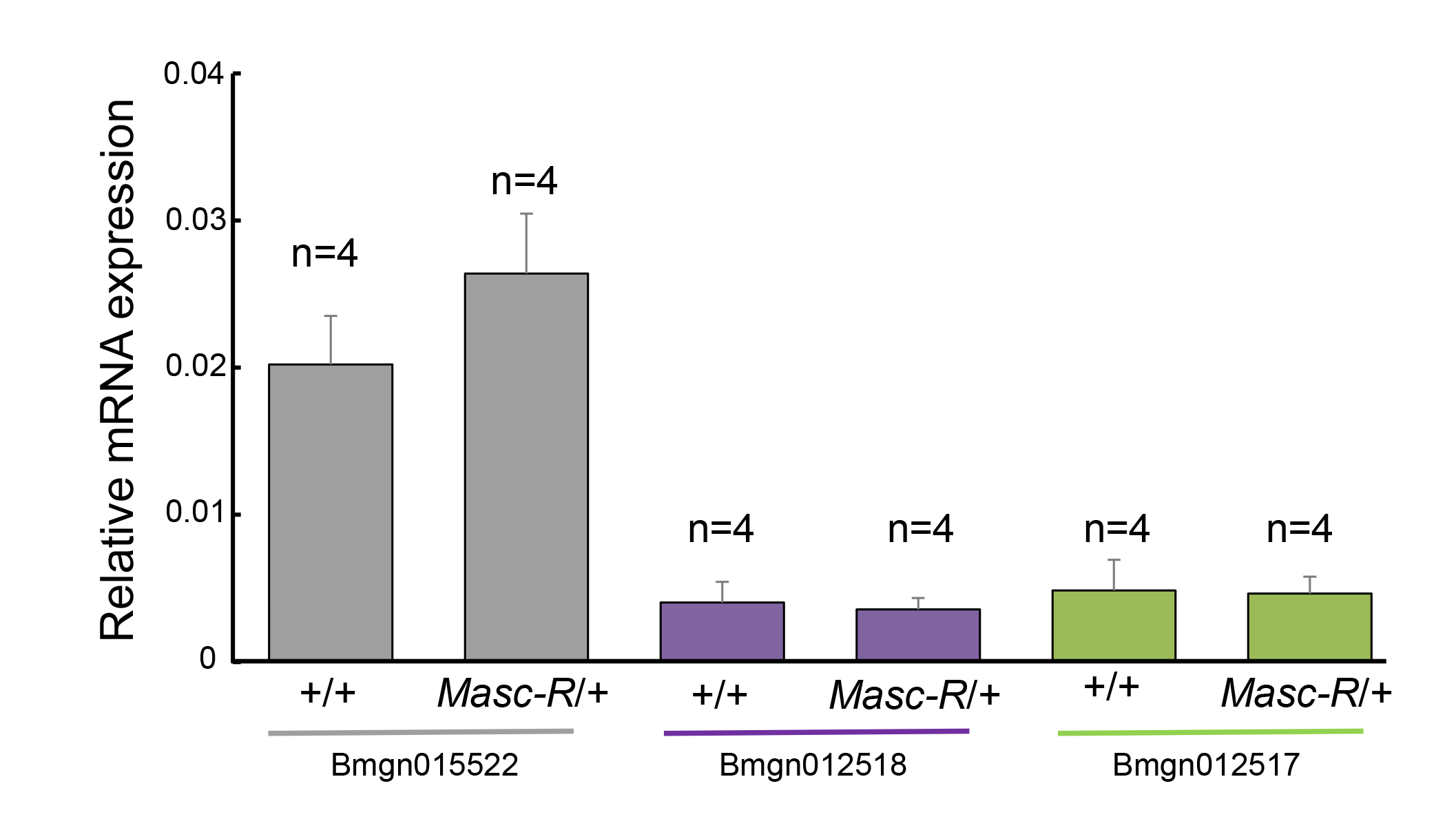

Supplement: S5 Fig — qRT-PCR was performed to quantify mRNA levels of Bmgn015522, Bmgn012518, and Bmgn012517 genes at L1D1. EF2 was used as an internal standard. Error bar: SD. (TIF) [file pgen.1006203.s007.tif]

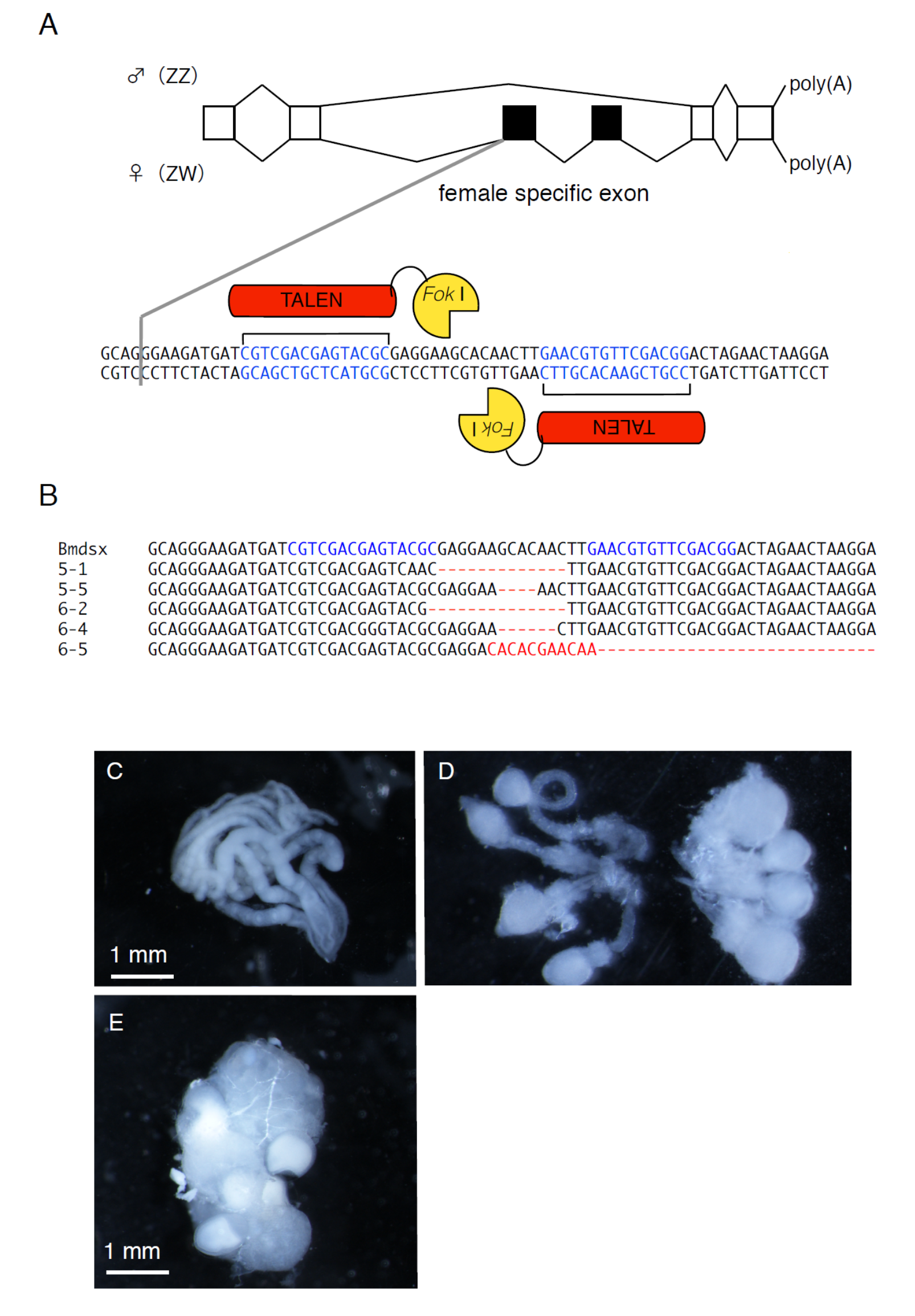

Supplement: S6 Fig — Knockout silkworms were generated using transcription activator-like effector nuclease (TALENs), as described previously [60, 61]. mRNAs encoding a transcription activator-like effector nuclease (TALEN) that targeted Bmdsx were injected into eggs, and hatched larvae (G0 animals) were subjected to the phenotypic analysis. (A) Target site of TALENs within female specific exon of Bmdsx. Splicing patterns of Bmdsx gene from male and female are shown. Exons are described by box, and female specific exons are in black. TAL effector-binding sequences within the female-specific exon 3 are shown in blue. (B) Variation of sequences around the TALEN target site from two G0 female lines (No. 5 and 6). Molecular sexing of G0 animals was determined by PCR using W chromosome RAPD markers, Musashi. The native sequence is shown at the top of the alignment (Bmdsx). Deleted nucleotides are shown as dashed line in red. 6–5 contained unknown inserted sequences (red) and lacked approximately 1 kbp of sequence downstream of the target site. The ovaries of the day-5 fifth instar larvae of normal females (C) and G0 females with mutations in BmdsxF (D). (E) The abnormal globular tissue observed in the ovary of the day-12 G0 female pupa. (TIF) [file pgen.1006203.s008.tif]
